# Multiplex enzymatic synthesis of DNA with single-base resolution

**DOI:** 10.1126/sciadv.adi0263

**Published:** 2023-07-07

**Authors:** Damiano Verardo, Beatrice Adelizzi, Daniel A. Rodriguez-Pinzon, Nicolas Moghaddam, Emma Thomée, Tessa Loman, Xavier Godron, Adrian Horgan

**Affiliations:** DNA Script, 67 Avenue de Fontainebleau, 94270 Le Kremlin-Bicêtre, France.

## Abstract

Enzymatic DNA synthesis (EDS) is a promising benchtop and user-friendly method of nucleic acid synthesis that, instead of solvents and phosphoramidites, uses mild aqueous conditions and enzymes. For applications such as protein engineering and spatial transcriptomics that require either oligo pools or arrays with high sequence diversity, the EDS method needs to be adapted and certain steps in the synthesis process spatially decoupled. Here, we have used a synthesis cycle comprising a first step of site-specific silicon microelectromechanical system inkjet dispensing of terminal deoxynucleotidyl transferase enzyme and 3′ blocked nucleotide, and a second step of bulk slide washing to remove the 3′ blocking group. By repeating the cycle on a substrate with an immobilized DNA primer, we show that microscale spatial control of nucleic acid sequence and length is possible, which, here, are assayed by hybridization and gel electrophoresis. This work is distinctive for enzymatically synthesizing DNA in a highly parallel manner with single base control.

## Teaser

Spatial enzyme-mediated nucleotide addition to immobilized DNA primers permits fabrication of DNA microarrays on glass substrates.

## INTRODUCTION

Enzymatic DNA synthesis (EDS) has the potential to be faster and give higher-purity DNA with none of the drawbacks (*1*, *2*) of the conventional phosphoramidite DNA synthesis method first proposed by Beaucage and Caruthers (*3*). Instead of harmful chemicals and flammable solvents, it relies on mild aqueous conditions and an enzyme called terminal deoxynucleotidyl transferase (TdT). Unusually for a polymerase, TdT does not need a template and is able to complex a broad range of divalent cations (e.g., Co^2+^) to lower the p*K*_a_ (where *K*_a_ is the acid dissociation constant) of the OH moiety at the 3′ end of a DNA primer (*4*). This facilitates nucleophilic attack on the 5′-phosphate of an incoming nucleotide to generate an extended primer and one molecule of pyrophosphate as a by-product. Although addition is usually spontaneous, the composition of the DNA primer; the presence of secondary structure; and the position, shape, and size of any modifications on the nucleotide may all influence the rate at which they are added (*5*–*8*). To ensure that addition occurs unfettered, a denaturing agent may be used to lower the melting temperature of the primer and the enzyme modified to enable its operation at elevated temperature (*9*).

Under optimum conditions, EDS coupling times are faster than in phosphoramidite synthesis (*10*), and left to itself, TdT will add nucleotides to the DNA primer unchecked (*11*, *12*). Different strategies have been proposed to prevent polycondensation to enable custom oligonucleotide synthesis (see fig. S1). One approach uses the enzyme to block further primer extension until a labile linker, positioned between the enzyme and the nucleotide, is cleaved in a subsequent step to detach the enzyme from the primer (*13*). An advantage of this route is fast kinetics without the need for enzyme engineering [because the nucleotide has a 3′-unblocked OH identical to a native deoxynucleotide triphosphate (dNTP) and conjugation ensures a high local concentration of the substrate]. A potential drawback is that it leaves a scar on the nucleobase. This has led others to suggest a hybrid strategy where the enzyme is attached through the terminal phosphate of the nucleotide, and polyaddition is prevented by a reversible terminator at the 3′ position (*14*).

Reversible terminators are 3′ blocking moieties that can be converted when needed to extendable 3′-OH (*15*–*18*). They can be used to exert single-base resolution, but the choice is quite restricted once stringent limits are placed on deblocking speed and efficiency. Furthermore, they need to be handled carefully as they may be good nucleophiles (*19*), and the wrong conditions may provoke side reactions such as deamination and the formation of adducts (*20*, *21*). Moreover, a necessary development step before their implementation is the screening of thousands of TdT variants to find one that accepts the 3′ blocking group, which can be a laborious iterative process (*22*).

A third way to wield control over the sequence has been called “free running enzymatic DNA synthesis” (*23*). This de novo DNA synthesis method modulates conditions to limit polycondensation to a minimum and is most suited to DNA data storage and related applications (*24*). One of the strategies proposed uses apyrase to compete for nucleotides and convert them to less TdT-active monophosphate and diphosphate analogs (*25*). Another uses reversible caging of Co^2+^ to regulate TdT activity and provides the only demonstration to date of multiplexed EDS (*26*). While notable, the number of synthesis cycles performed (8) and the number of individual sites of DNA synthesis (12) were relatively modest, and the approach lacked single-base control.

In this work, we report the first multiplex EDS demonstration with single-base control. We also show a longer length than nonmultiplexing studies that showed single-nucleotide addition. With optimization, the method could provide custom DNA for applications such as protein engineering. Microarray DNA is the cheapest available source of synthetic DNA but is currently made using conventional phosphoramidite chemistry (*27*). Here, we have used silicon microelectromechanical system (MEMS) piezoelectric printheads to deposit enzyme and nucleotides with 3′ reversible terminator groups site-specifically onto a substrate. This is radically different from the industrial-scale inkjet DNA synthesis using phosphoramidites commercialized by Agilent and Twist Biosciences (*28*, *29*). Inspired by the POSaM platform (*30*–*32*), we have used low-cost, off-the-shelf components and built a benchtop instrument for de novo synthesis of DNA microarrays in research laboratories. The benchtop advantage EDS affords is that it removes any requirement for anhydrous solvents, dry atmosphere, airtightness, and inert gas, i.e., glove box conditions (*33*, *34*).

## RESULTS

### The inkjet platform

**Fig. 1.**
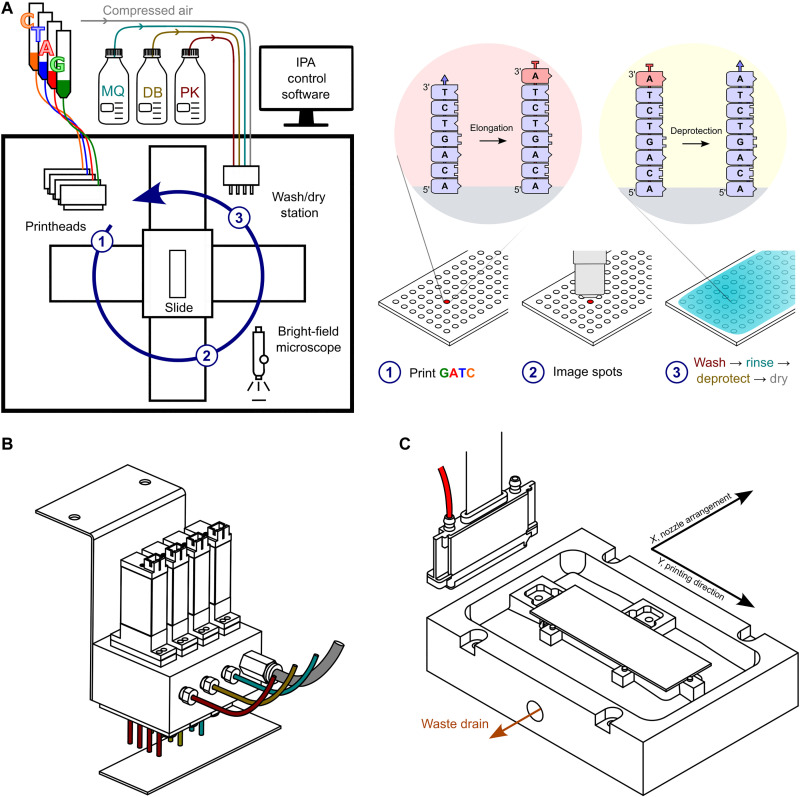
The EDS inkjet platform. (**A**) Schematic of the printer and the EDS synthesis cycle. The four printheads (600-dpi resolution), custom slide holder, and custom washing/drying station are enclosed in a transparent box that protects against dust. The humidity inside the enclosure is controlled at ~65% RH using a Cellkraft PD-10 (not shown) to prevent ink drying at the printhead or on the substrate. Each printhead (1) deposits in a spatially addressable manner an ink containing a 3′-ONH_2_–blocked nucleotide (red) and TdT enzyme onto a glass microscope slide on which is a 5′-end-immobilized DNA primer (5′-surface-DNA-3′-OH). After bright-field imaging with a microscope (2) to determine droplet properties (presence, size, position, and shape), the substrate is moved in its holder to a wash/dry station (3). One of the wash fluids is used to neutralize the enzyme reaction. Another converts the blocking group (red) on the 3′ end of the extended DNA primer (5′-surface-DNA_n+1_3′-ONH_2_) to a freely extendable (blue) 3′-OH (5′-surface-DNA_n+1_3′-OH) so another cycle of elongation can be performed. (**B**) Side view of the washing and drying station. The wash head has three rows of four nozzles for deprotection buffer (DB), proteinase K wash buffer, and Milli-Q water. A fourth row is dedicated to slide drying and is connected to a compressed air source. (**C**) View from above of the slide holder. The slide holder is mounted on two *XY* direct drive stages. It is made from polytetrafluoroethylene and uses two stainless steel springs to push the glass slide against three metal retainer posts. The slide is suspended above a trough that has a waste drain attached to a peristaltic pump. The pump is switched on during slide washing.

The layout of the inkjet synthesis platform and the relationship with the EDS synthesis cycle are depicted in Fig. 1. To spatially control the 3′ extension of DNA primers on a substrate, four thin-film silicon MEMS printheads with 600 dots per inch (dpi) native resolution were used to print inks containing enzymes and nucleotides (A, C, G, and T). In the ink were also additives to prevent evaporation, reduce secondary structure, and scavenge adventitious agents that might cap the 3′-aminoxy blocking group (e.g., formaldehyde) (*35*). Reproducible and reliable printing without satellite spots was assured by active control of the meniscus pressure at the nozzle plate and by adjusting the ink’s rheology into a range identified by a plot of Ohnesorge number against Reynolds number (*36*). A lower viscosity ink was chosen to avoid the possibility of negatively affecting enzyme activity (*37*), and tests were done to ascertain whether TdT activity was altered by the choice of detergent and by jetting, given that ejection occurs at several meters per second and involves high shear (*38*–*45*). Latency, a negative behavior of ink after a period sitting idle in the printhead, was reduced by nozzle tickling, periodic dummy printing, and increasing the humidity in the enclosure, which also helped to ensure that the picoliter droplets deposited on the substrate did not evaporate during incubation postprinting.

Automation of the synthesis cycle involved moving a substrate (a standard microscope slide coated uniformly with a cleavable DNA primer) under the printheads to an imaging station, then to a wash station, and then back to the starting (home) position (Fig. 1A). Slide washing was initiated after incubating the enzyme and nucleotide for 5 to 10 min during which time the slide was imaged, and the positions and sizes of the printed spots were recorded. Compressed air was used to convey liquids to a wash head equipped with four electronic valves (Fig. 1B), each fluidically connected to a row of four nozzles and under the control of an Arduino board interfaced with an in-house software called the Inkjet Printer App (IPA), written in C#. To remove wash liquids, the substrate was mounted on a holder with trough and liquids were emptied under IPA control via a peristaltic pump (Fig. 1C). Slide drying was performed after slide washing using a serpentine motion.

The setup, as described, proved to be robust and effective. However, some changes would be needed to lower instrument cost further while making synthesis faster and reagent use more efficient. For instance, a much simpler and cheaper means of controlling humidity would pull moisture from a benchtop humidifier into the enclosure using a fan activated using a proportional–integral–derivative (PID) controller connected to a humidity probe in the enclosure. No effort was made in this work to control temperature at the slide surface or within the Perspex enclosure, but one option would be to use a heated slide cover and apply the cover when incubating. From a washing point of view, more nozzles would give a more uniform dispense of the liquid, and an air knife would allow the liquid to be swept off the slide in a single motion. We also note that some intended improvements might have drawbacks. For instance, a thermally conductive gold or silicon substrate might facilitate warming during incubation but might complicate imaging.

### Surface chemistry

DNA may be purchased with various 5′ modifications (e.g., amino and thiol) and covalently immobilized to silanized or polymer-coated substrates for use as a primer for EDS. In the present work, a 5′-DBCO (dibenzocyclooctyne) primer with a photocleavable group (see fig. S2) was incubated with an azide-modified glass substrate at room temperature in aqueous conditions (see Materials and Methods). The copper-free Click SPAAC (strain-promoted azide–alkyne cycloaddition) reaction (*46*) was observed to be very fast (<10 min) in agreement with the literature (*47*) and resulted in a uniform layer of DNA as judged by signal intensity (see fig. S3). While incubation with different DBCO-modified small molecules and oligomers modified the contact angle, suggesting that there were residual azide groups remaining on the surface, quenching was not used in the present work.

**Fig. 2.**
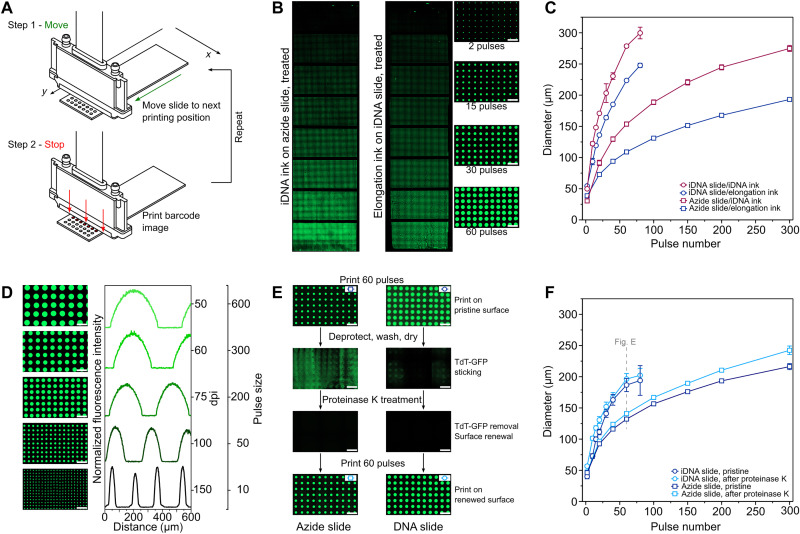
Printing of inks. (**A**) The “move-stop-print” principle. (**B**) Change in spot size. Left: Epifluorescence microscope image of FAM-labeled DNA spots after printing of DNA ink on an azide surface, coupling, and automated slide washing. The nozzles were pulsed 2, 20, 40, 60, 100, 150, 200, and 300 times from top to bottom. Center: Image of an array printed with an ink formulated to allow elongation of a uniform DNA surface. Nozzles were actuated 2, 10, 15, 20, 30, 40, 60, and 80 times from top to bottom. After incubating, nonprinted areas were manually capped with TdT + dideoxyadenosine triphosphate (ddATP). Automated slide washing was then performed to remove the 3′-ONH_2_ protection; then, end-labeling was done manually with TdT + FAM-labeled ddATP. Right: Close-up of the right (center) array. (**C**) Wetting versus surface type. A different number of droplets was printed in eight sections on the substrate and then imaged wet. To allow imaging, the elongation ink contained 50 μM fluorescein. The reported mean droplet size is for two printed slides. (**D**) Control over pitch and droplet size. Images and normalized profiles obtained for DNA ink on an azide slide using (top to bottom) 50 dpi: 600 pulses, 60 dpi: 300 pulses, 75 dpi: 200 pulses, 100 dpi: 50 pulses, 150 dpi: and 10 pulses. (**E**) Protein adsorption. Images of an azide slide (left) and a DNA slide (right) after (top to bottom) first printing (60 pulses) with the TdT–green fluorescent protein (GFP) elongation ink; then automated deprotection and water washing; then automated proteinase K treatment, deprotection, and water washing; and then printing elongation ink again (60 pulses). Slide washing leads to nonuniform adsorption of TdT that can be removed by a proteinase K wash. (**F**) Effect of proteinase K treatment on droplet size. The effect on droplet size is negligible for the DNA surface. Scale bars, 500 μm.

To determine the relationship between ink and surface and to examine the influence of synthesis parameters more easily, a new “move-stop-print” line-by-line printing process controlled by the IPA was developed (Fig. 2A). It comprised a series of rapid (<500 ms) stepwise movements in the *Y* direction in between which nozzles in the perpendicular (nozzle row) direction (*X*) were actuated while the substrate was stationary. Each nozzle actuation deposited a ~3-pl droplet on the substrate below the nozzle location. Images resembling one-dimensional (1D) barcodes were used to determine which nozzles were fired and how many times the addressed nozzles were fired at each stop (see fig. S4). Among its advantages, the method allows the deposition on the substrate of any drop volume in precise locations without repeated back and forth movements and printing on the fly based on encoder position. It is also more time efficient for large droplet (i.e., wet)/spot (i.e., dry) sizes, which are easier to image and afford DNA spots with higher mass for faster, simpler sample analysis without a need for polymerase chain reaction (PCR) amplification and next-generation sequencing (NGS) library preparation.

Printing tests were done with two jettable inks, one containing a 5′-DBCO primer (here called “DNA ink”) and the other having an enzyme and a 3′ blocked nucleotide (here called “elongation ink”). The first tests aimed to show DNA coupling and DNA elongation for different spot sizes. Both inks were printed at 75 dpi (see fig. S5) on the appropriate surface (an azide-modified microscope slide for DNA ink and an azide slide modified with a uniform layer of a 5′-DBCO primer for elongation ink) using a different number of droplets in each section of the slide (Fig. 2B). Imaging was done with an epifluorescence microscope after automated slide washing and drying. An additional step of TdT-mediated end-labeling with 6-Carboxyfluorescein (6-FAM)–labeled dideoxynucleotide was performed before imaging in the case of the elongation ink (see Materials and Methods). In both cases, the dry spots were bright, regular in shape, and uniformly distributed (see fig. S6). Both inks were also printed on both surfaces and imaged wet without any further treatment (see fig. S7). The droplet sizes are reported in Fig. 2C. Using the same drive waveform (*48*), the DNA ink gave larger droplets than the elongation ink, and both inks spread more on the hydrophilic DNA surface than on the hydrophobic azide surface.

Having shown control over the droplet size, trials were done to see whether the separation between droplets (pitch) could be varied at the same time as size by activating subsets of nozzles in the *X* direction (nozzle row) (Fig. 2D). Printing was done on the azide surface using DNA ink, and the printed droplets were imaged (see fig. S8). The number of pulses (droplets ejected) was varied from 10 to 600, while the number of nozzles activated was changed to cover the range from 150 to 50 dpi (in the *X* direction). Collectively, the results are useful to determine an experimentally convenient spot size and density for subsequent EDS tests (60 pulses and 75 dpi). They also allow estimation of the maximum sequence diversity that might be achievable using the formulated inks and a hexagonal close-packed arrangement (~1 M per slide) versus the square pattern used here (see fig. S5).

As a final printing test, an elongation ink at pH 6.0 containing green fluorescent protein (GFP)–labeled TdT was printed on the azide and DNA surfaces to gauge the extent of protein adsorption (Fig. 2E). TdT [isoelectric point (pI) ~ 6.5, melting transition temperature (*T*_m_) ~ 42°C] proved to be a more difficult protein to print than model proteins bovine serum albumin (pI ~ 4.5, *T*_m_ ~ 63°C) or lysozyme (pI ~ 11.4, *T*_m_ ~ 72°C), and this led us to suspect that protein adsorption might be an issue. It was observed that the droplets of the GFP-TdT elongation ink are smaller than in Fig. 2C. We attribute this to the additional presence of GFP (pI ~ 6.2, *T*_m_ ~ 78°C) and its coupling to an earlier TdT mutant. A second observation was that protein adsorption was nonuniform because of the method of slide washing and was more severe on the azide surface than on the DNA surface. To counteract this problem, a proteinase K treatment was tried and found to be an effective solution for renewing the surface in both cases (see fig. S9). Little evidence was found of proteinase K (or Tween 20) adsorption on the DNA surface, as judged by a second print and by comparison of the size and shape of the droplets with the first (Fig. 2F). Most importantly, the droplet arrays on the surface after proteinase K washing were regular.

### Ink formulation

Inks intended for use in inkjet printers should be degassed to avoid the formation of air occlusions, should be filtered to avoid nozzle blocking by debris, and should be inherently stable. To circumvent nozzle “drop out” caused by blockages, nozzles can be held in reserve, but if clogging is extensive, then the only viable solution is to perform repeated priming and purging of the printhead during which time the printer is inactive. In the case of inkjet-enabled multiplex EDS, an additional requirement is that the ink remains active during synthesis (>24 hours if a cycle takes 10 min and a run has 150 cycles). Furthermore, it would be beneficial on cost and convenience grounds if the ink remained active for multiple runs given that the inner volume of a printhead will exceed the volume printed in a typical synthesis run by one or two orders of magnitude.

**Fig. 3.**
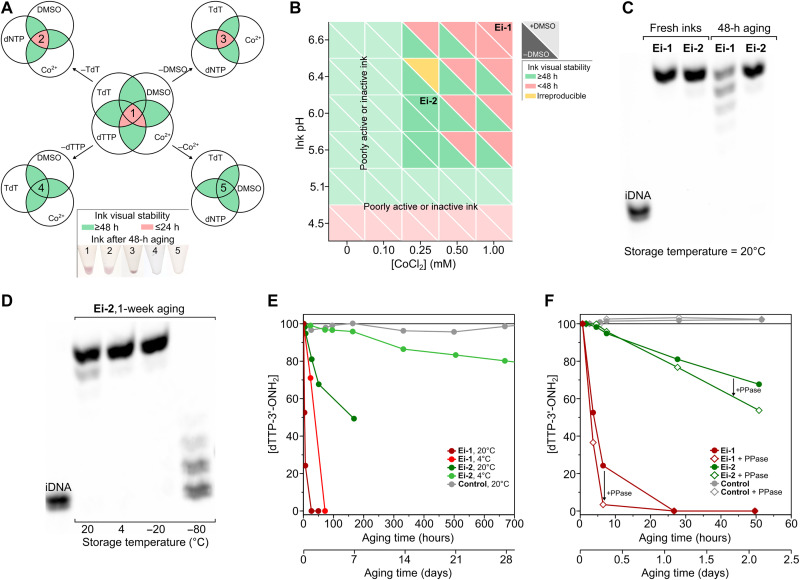
Ink stability and activity. (**A**) Stability of initial elongation ink (Ei-1). Venn diagrams showing stable (green) formulations and unstable (red) formulations, giving precipitate when aged 48 hours at 20°C. (**B**) Stability of elongation inks as a function of pH, [CoCl_2_], and dimethyl sulfoxide (DMSO). Green denotes stable ink; red denotes inks that precipitate in ≤48 hours at 20°C. Ei-2 was selected as ink for this work on the basis of its stability and activity. It has 500 μM dTTP-3′-ONH_2_, 20 μM TdT, 0.25 mM CoCl_2_, 15% (v/v) DMSO, 50 mM *O*-benzylhydroxylamine·HCl, 10% (v/v) glycerol, 0.05% (v/v) Tween 20, 2.5 mM tris-HCl, 40 mM NaCl, 0.5 mM Hepes, 0.5 M cacodylic acid, and a final pH of 6.0 (measured in the absence of DMSO). (**C**) Ink activity of select inks. Polyacrylamide gel electrophoresis (PAGE)–urea gel of manually synthesized sequence TTT-TTT-T. Synthesis was performed with Ei-1 and Ei-2 inks freshly prepared and aged 48 hours at 20°C. (**D**) Effect of ink cooling. PAGE-urea gel of manually synthesized TTT-TTT-T with Ei-2 aged for 1 week at 20°, 4°, −20°, and −80°C. (**E**) Degradation of dTTP-3′-ONH_2_ according to ion exchange high-pressure liquid chromatography with ultraviolet detection (IE-HPLC UV). Results for ink Ei-1 and Ei-2 as a function of the temperature (4° and 20°C) and aging time. Control ink does not contain CoCl_2_ or TdT. (**F**) Effect of pyrophosphatase. IE-HPLC UV measurement of [dTTP-3′-ONH_2_] for Ei-1 and Ei-2 inks in the presence and absence of inorganic pyrophosphatase (PPase). Control inks with and without PPase do not contain CoCl_2_ or TdT.

Initial results for EDS ink stability were not promising. A precipitate formed in less than 12 hours, and its pink color suggested the formation of a cobalt complex. Components were therefore removed one by one from the elongation ink (“Ei-1”) to understand their contribution (Fig. 3A). This confirmed the involvement of cobalt and also the importance of the nucleotide. With an understanding of which components were most detrimental, we embarked on a study of pH and cobalt concentration. The pH of the ink was lowered to exclude the possible binding of the his-tag on the protein (TdT), which is most efficient at a slightly basic pH (~7.5 to 8). The presence or absence of his-tag, however, had no bearing on ink stability, but the pH and concentration of cobalt had a pronounced effect (Fig. 3B). Lowering both markedly improved stability.

Having found a more physically stable formulation (“Ei-2”), tests were done to determine if there was a concomitant improvement in activity. According to gel electrophoresis, activity was much improved and unchanged over 48 to 72 hours (Fig. 3C), a period sufficient for at least two or three long synthesis runs. Cooling the ink extended the lifetime of the ink further (Fig. 3D) and could help in other ways. The operating temperature of most piezo-silicon MEMS printheads is between 5° and 40°C, so printing of cooled ink is feasible if the substrate is warmed. The added benefit of cooling is that, at lower temperatures, ink viscosity will be higher, meaning a reduced need for viscosity modifier, with the former advantageous for jetting and the latter for ink activity (*37*). We also observed that the presence of glycerol and dimethyl sulfoxide (DMSO; added to modify viscosity and hinder secondary structure formation, respectively) allowed the ink to be stored below −18°C in liquid form. This raises the possibility of ink transport without lyophilization and separation of components into hermetic compartments of an expensive microfluidic cartridge.

With the advantages of cooling established, we wanted to see whether it was possible to develop an ink that is room temperature stable. The starting point was to understand the behavior of the most labile species, which was hypothesized to be the nucleotide. The terminal (γ) phosphate of a nucleotide is susceptible to hydrolysis, and at room temperature, there may be decomposition to the corresponding diphosphates and monophosphates at a rate of ~1% over 6 weeks (*49*, *50*). High-performance liquid chromatography (HPLC) confirmed the benefit of lowering the pH, cobalt concentration, and temperature (Fig. 3E) but still revealed a clear difference with ink lacking cobalt or TdT (“Control’). The degradation was not therefore simply due to hydrolysis.

During these studies, two grades of nucleotide became available, and precipitation was observed to be faster for the lowest grade containing more pyrophosphate (see fig. S10), which is known to form insoluble precipitates with cobalt (*51*). Inorganic pyrophosphatase was therefore added to the inks, which led to visual stability even for ink Ei-1 (Fig. 3F). Unfortunately, it sped up the degradation of the nucleotide according to HPLC. To explain these results, it was hypothesized that the pyrophosphatase was encouraging a forward reaction involving the α-phosphate of the nucleotide and was generating pyrophosphate as a by-product with the reaction mediated by TdT and cobalt. Liquid chromatography–mass spectrometry (LC-MS) was therefore conducted on the unmodified ink, with and without cobalt, to analyze the mass change of the nucleotide. A rapid degradation in the presence of cobalt was apparent but not in the metal ion’s absence, and a new peak emerged with a mass corresponding to a species at 410 mass/charge ratio that is probably an adduct of glycerol (see fig. S11). Tests using the purer nucleotide (performed with and without TdT), cobalt, and glycerol provided additional support for the proposed nonhydrolysis pathway (see fig. S12). As noted by Schaudy *et al.* (*5*) and Baiga *et al.* (*52*), TdT can prime off non-nucleic acid primers such as hexaethylene glycol. Hence, to preserve activity and stability, the ink should lack endogenous pyrophosphate or species that could lead to its generation by acting as a pseudo-DNA primer. If the ink does contain pyrophosphate or a potential precursor, then the ink should not contain inorganic pyrophosphatase. Other solutions are to screen enzymes for activity against any non-nucleic acid primers and to split ink components (e.g., TdT and cobalt) by compatibility and then mix them just before printing or print them separately on the slide surface.

### Spatial EDS with single-base control

**Fig. 4.**
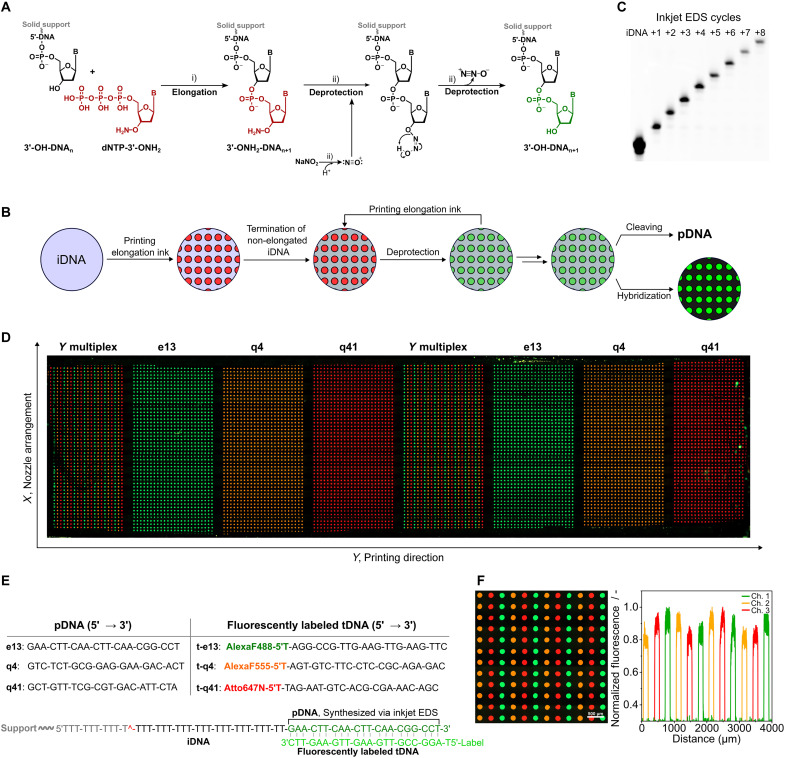
Spatial EDS using inkjet dispensing. (**A**) EDS principle and deprotection mechanism. Steps: (i) TdT-mediated elongation of the 3′-OH-DNA primer (iDNA) with the reversible terminator dNTP-3′-ONH_2_ to afford 3′-ONH_2_-DNA_n+1_ and (ii) nitrosonium-mediated deprotection to 3′-OH-DNA_n+1_. (**B**) Inkjet EDS method showing single-base control. Elongation ink containing dTTP-3′-ONH_2_ (Ei-2) was printed onto a DNA-covered surface and left to incubate 10 min at 20°C; then, nonprinted areas were capped by incubation for 5 min with a ddATP elongation ink. The slide was then washed consecutively for 1 min with a buffered solution of proteinase K, 1 min with water, 3 min with a DB containing 0.7 M NaOAc and 1.0 M NaNO_2_ at pH 5.2, and 1 min with water. All liquids were at room temperature and dispensed under IPA control as was slide drying with compressed air. (**C**) PAGE-urea gel displaying single-base length control via inkjet EDS. The image shows the increase in length of a poly(T) sequence synthesized via 1 to 8 cycles of inkjet EDS. The DNA was enzymatically end-labeled with ddATP-FAM for 10 min, photocleaved from the substrate in 0.1× phosphate-buffered saline (PBS) at λ365 nm (15 min). (**D**) Sequence and array-wide spatial control. Epifluorescence microscope image of a whole slide after competitive hybridization of three complementary fluorescent target oligonucleotides (t-e13, t-q4, and t-q41) to three EDS-printed probe sequences (e13, q4, and q41). (**E**) EDS synthesized probes and their complementary targets. EDS starts with a DNA primer bound via its 5′ end. The primer contains a UV-cleavable group for release postsynthesis and analysis via gel electrophoresis. pDNA, printed DNA; tDNA, target DNA; iDNA, initiator(/primer) DNA. (**F**) Close-up of a section where the three sequences were multiplexed side by side in the *Y* direction (perpendicular to the direction of slide movement) and the related fluorescence profiles (channel 1, enhanced GFP; channel 2, DsRed; channel 3, Cy5).

To carry out EDS and add dNTP-3′-ONH_2_ nucleotides one by one to a DNA primer as shown in Fig. 4A, we used the deprotection buffer (DB) of Hutter *et al.* (*19*), a pH-adjusted solution of sodium nitrite and sodium acetate. The mechanism of 3′-ONH_2_ (aminoxy) deblocking has not been elucidated to our knowledge, but the reaction likely proceeds by nucleophilic attack by the 3′-aminoxy on the nitrosonium cation (*53*) followed by cyclic elimination of nitrous oxide (N_2_O). The reaction speed and specificity are due to the enhanced nucleophilicity of the 3′-aminoxy group resulting from an alpha effect and its lower p*K*_a_ (~6) compared to primary amines. Unwanted side reactions are a possibility, but as in the case of phosphoramidite synthesis, their incidence depends on the degree of optimization (*29*). Depurination can be suppressed by adjustment of pH and addition of salts and other additives (*21*), while deamination resulting from nonspecific nitrosation of the base takes place on a time scale significantly longer (4300 times) than the time required to cleave 98% of the 3′-ONH_2_ groups (*19*). Furthermore, both issues can be avoided entirely by switching to a non-nitrosation method such as reductive cleavage (*54*) or through base protection, which is a standard practice in conventional DNA synthesis. We chose to exemplify high-multiplex EDS with single-base control using the simplest synthesis cycle and choice of reagents. Our cycle has only two reaction steps: nucleotide coupling under mild conditions and 3′-ONH_2_ deprotection with a short exposure (<3 min) to nitrous acid. By comparison, DNA synthesis using phosphoramidites has five reaction steps: deblocking (detritylation), coupling, oxidation, capping, and removal of base protecting groups; most of which use an organic solvent.

To demonstrate inkjet EDS and single-base control, a single-enzyme ink containing 3′-deoxythymidine 5′-triphosphate (dTTP)–3′-ONH_2_ was printed multiple times onto a glass slide with a uniform covering of DNA using the move-stop-print method. DNA was not printed because there were insufficient printheads to print both the DNA ink and the four enzyme-containing inks (A, G, C, and T) needed for a subsequent real-sequence demonstration. In addition, enzyme adsorption was previously shown to be worse for an azide surface. A medium spot size (15 pulses) and spot density (75 dpi; ~16,000 synthesis sites/slide) were chosen, taking into consideration prior results and a desire to make monitoring of the printing process and analysis of the results straightforward. First-cycle elongation was followed by capping of DNA primer in nonprinted areas through incubation with an elongation buffer containing TdT and dideoxyadenosine triphosphate (ddATP). After this step, and after each subsequent round of printing (60 pulses to allow for any imprecision and ensure coverage of the sites of synthesis), the slide was washed with proteinase K, water, DB, and water again before drying. Figure 4C shows the change in the length of the DNA with each cycle of printing (coupling) and washing (deprotection). The number of sections printed on was reduced by one each cycle to afford eight printed sections, where the DNA length differs by one base. The DNA was subsequently imaged on the slide (see fig. S13) and then released from the solid support by photocleaving at λ365 nm for length separation by polyacrylamide gel electrophoresis (PAGE)–urea gel electrophoresis. This yielded a ladder with single bands with one nucleotide difference in length.

Given these promising results, three real sequences of 21 bases with a similar CG content and melting temperature (see fig. S2) were next synthesized in duplicate in eight sections on a DNA-coated glass slide. One sequence was synthesized per section except for sections 1 and 5, where the three sequences were synthesized side by side in rows for a first, very simple demonstration of multiplexing. Competitive hybridization of complementary targets, each labeled with a different dye, was then used to reveal the locations of the sites of synthesis and the positions of the three sequences (Fig. 4D). Excellent sequence discrimination, spot uniformity, signal to noise, and signal homogeneity are evident (see fig. S14), which are strong indicators of well-controlled single-base extension and reproducible printing. Sequence purity was confirmed by gel electrophoresis, with each lane showing a single intense band from which purity and cycle efficiency (~99%) were estimated (see fig. S15).

### Multiplex EDS

**Fig. 5.**
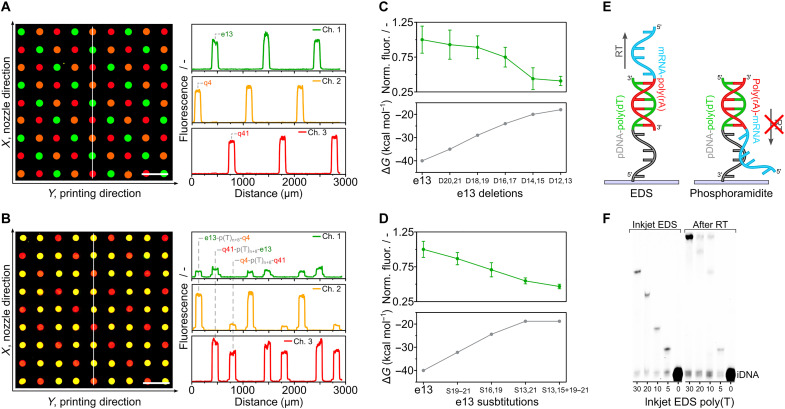
EDS multiplexing. (**A**) 2D multiplexing. The three sequences of Fig. 4 were arrayed in an *XY* pattern, requiring control of selected nozzles in the nozzle row (*X* direction). Their locations were revealed by competitive hybridization. (**B**) 3D multiplexing. The following three 50-mer sequences [e13-poly(T)_n+8_-q4, q4-poly(T)_n+8_-q41, and q41-poly(T)_n+8_-e13] were synthesized separately and in an *XY* pattern, and their locations were revealed by competitive hybridization with two target DNAs per synthesized strand. (**C**) Determination of synthesis errors (deletions) by hybridization. Fluorescence intensity after hybridization to e13 probes deliberately synthesized with double deletions. (**D**) Determination of synthesis errors (substitutions). Fluorescence intensity after hybridization to e13 probes deliberately synthesized with substitutions. (**E**) The EDS advantage in spatial transcriptomics. EDS proceeds in the 5′ to 3′ direction, allowing poly(T) tails to capture mRNA and be reverse transcribed unhindered. (**F**) Capture of mRNA and reverse transcription (RT) to cDNA. Poly(T) surface probes were synthesized with different lengths and then incubated with a short mRNA of sequence 5′-UACACGUUGUCUAUCGCCUU(30A)-3′ (see fig. S2). After reverse transcription and dehybridization, the resulting cDNA was end-labeled and cleaved from the solid support, and the increase in length was confirmed by gel electrophoresis..

Having used the move-stop-print method to demonstrate control over spot size and pitch (density) and then shown microscale EDS of real sequences, we sought to do a true demonstration of multiplexing. Instead of printing the three sequences horizontally (in the *X* direction) parallel to each other, they were juxtaposed in both the *X* and *Y* directions (i.e., a 2D configuration) (Fig. 5A). They were also printed in the *Z* direction with one sequence on top of another to demonstrate that lengths above a typical PCR primer (18 to 30 bases) are attainable. Multiplexing was limited to just three unique sequences because of the filter set of the microscope and the desire to keep the analysis as quick and as direct as possible without PCR. Figure 5B shows the result of competitive hybridization and confirms that the three sequences had the correct *XYZ* coordinates (see also fig. S16). Gel electrophoresis (see fig. S17) corroborates the difference in length of the *XY* and *XYZ* probe sequences of fig. S2.

To see whether hybridization could be used to identify major and minor synthesis errors caused by printing imprecision, for example, a droplet not being printed in an assigned location at a given cycle, the three probes were deliberately synthesized with mismatches and deletions and hybridized. Figure 5C shows the example of double deletions and compares signal with theoretical calculations of the hybridization free energy (Δ*G*) according to IDT’s OligoAnalyzer (“Hetero-dimer analysis”). As anticipated (*29*), the hybridization signal varied inversely with the number of deletions on the 5′-terminus while translation of deletions to the middle of the probe exerted a greater effect than deletions at the extremities because of the reduced degree of complementarity (see fig. S18). Substitutions, which are less likely to be caused by printing errors but might occur because of poor slide washing, also had a significant impact on intensity (Fig. 5D), especially when situated near the interior of the probe (see fig. S19).

With DNA purity confirmed by hybridization and by gel electrophoresis, we set out to highlight one of the potential advantages of spatial EDS. A DNA primer was uniformly immobilized to a substrate via its 5′ end and on its 3′ end was inkjet synthesized a polythymidine [poly(T)] tail. As EDS proceeds in the 5′ to 3′ direction, the synthesized DNA, which could include a barcode, unique molecular identifier, and handle for amplification, was able to freely capture the poly(A) (polyadenylate) tail of a short mRNA molecule (Fig. 5E). Reverse transcription was then performed using the poly(T) tail as a primer to generate cDNA, which was end-labeled, cleaved, and analyzed by gel electrophoresis. Figure 5F shows the corresponding increase in DNA length (+20 bases), which can only happen if there has been mRNA capture (see fig. S20). By synthesizing in the 5′ to 3′ direction, spatial transcriptomics, which seeks to relate protein expression to 2D location in histological sections, is made easier. In the method of Stahl *et al.* (*55*), the transcriptome of only 1007 locations (occupying an area of ~41 mm^2^) was elucidated because the barcoded mRNA capture probes first had to be synthesized individually in the 3′ to 5′ direction using the phosphoramidite method by an external supplier. Then, they had to be arrayed on a surface in the opposite direction before tissue application. A much higher barcoding density (~10^6^ K/mm^2^) for subcell resolution was achieved using a bead array (*56*), but the oligos required had to be synthesized first on a phosphoramidite synthesizer, then immobilized to beads then extended via split-and-pool ligation, and then decoded through several rounds of hybridization and fluorescence imaging after transfer of the barcoded beads into the wells of a microfabricated substrate (*57*). Spatial EDS is potentially a much simpler way to diverse, and dense arrays of ordered barcoded probes with their 3′ poly(T) ends correctly orientated unhindered away from the surface for the capture and transcription of mRNA. Access to a printer in research laboratories would also allow researchers to modify the resulting cDNA, for example, by adding a common 3′ primer for sequencing.

## DISCUSSION

We report an advance in de novo DNA synthesis: spatial microscale EDS with single-base control. A printer equipped with four silicon MEMS piezo inkjet printheads has been shown to be able to deposit picoliter volumes of ink necessary for EDS with micron precision in preselected locations on a glass slide in which a DNA primer has been immobilized. Within each ink droplet on the surface and in every synthesis cycle, a template-free polymerase (TdT) adds a single nucleotide to the 3′ end of the DNA primer, with the risk of multiple additions precluded by a 3′ blocking group that is removed by washing of the slide. If required, the DNA can be cleaved enzymatically at the 5′ end postsynthesis to give the desired DNA without primer (*58*). We have shown that hybridization and gel electrophoresis can be used to confirm length and gauge sequence accuracy for an EDS synthesized microarray. Key issues such as ink stability have been identified and addressed to ensure reproducible results and ease of use. Similar to the benchtop PoSAM platform (*30*), our printer is simple and inexpensive, being assembled from off-the-shelf components. Unlike PoSAM, our printer permits control over spot size (mass) and offers far more than 9600 sites of synthesis, while it also eliminates the requirement of existing inkjet printers for glove box conditions. The cost of a phosphoramidite-synthesized oligo pool is estimated to be $1 per 10^3^ to 10^5^ base pairs (*59*). As we have shown, an EDS reaction cycle needs only micromolar quantities of reagent and can have fewer steps, which means fewer washes with water, which happens to be significantly less expensive than acetonitrile. Although an inkjet DNA printer should consume very little reagent once running and should generate relatively little waste, the storage and disposal of aqueous reagents is less onerous than organic reagents. Thus, we anticipate that the cost of an EDS oligo pool will be inferior. To achieve the longest lengths, highest density, and lowest error rates, further optimization of machine parameters and EDS reagents and protocols will be necessary. Looking forward, spatial EDS could, one day, become routine and commonplace in research laboratories and accelerate biological research by enabling researchers to iterate quickly with much greater flexibility than at present.

## MATERIALS AND METHODS

### Immobilization of DNA initiators on glass substrates and DNA cleavage

The 2D azide glass slides (PolyAn, Berlin, Germany) were mounted in a ProPlate one-well slide gasket from Grace Bio-Labs Inc. (Bend, OR, USA) and incubated with 5 μM 5′ DBCO-triethyleneglycol–modified oligonucleotide (Eurogentec, Belgium) in 0.1 M NaOAc and 0.5 M NaCl buffer (pH 4.5) at 20°C in a chamber at 70% relative humidity (RH). After 1 hour, the coupling solution was removed and the slide was rinsed with 1500 μl of 0.1 M NaOAc and 0.5 M NaCl buffer (pH 4.5), 5 × 1500 μl of phosphate-buffered saline (PBS), 50 ml of DB (1 hour), and 50 ml of Milli-Q (5 min), followed by immediate drying with compressed air. For DNA printing, the DNA primer ink (pH 4.5; measured without DMSO) was formulated in Milli-Q:DMSO [65:35 (v/v)] with 5 μM initiator/primer DNA (iDNA), 325 mM NaCl, and 65 mM NaOAc.

### Move-stop printing and automation

The 1D barcode images (see fig. S4) were generated using an in-house software called the Inkjet Printer App and printed according to the cycle and position on the array. The positions of the bars in the image determine the nozzles actuated in the nozzle row, and the lengths of the bars determine how many actuations (pulses) each nozzle makes. A 5-V trigger signal was used to initiate printing after each move of the translation stage.

### Ink studies

The ink [pH 6.6 (Ei-1) or pH 6.0 (Ei-2)] contained 10% (v/v) glycerol, 0.025% (v/v) Tween 20, 2.5 mM tri-·HCl, 40 mM NaCl, 0.5 mM Hepes, 0.5 M cacodylic acid, 50 mM *O*-benzohydroxylamine hydrochloride, 0.25 (Ei-2) or 1 (Ei-1) mM CoCl_2_, 500 μM 3′-ONH_2_-dNTP (N = A, C, G, T), 20 μM TdT, and 15% (v/v) DMSO. Inks for stability tests were stored in plastic Eppendorf tubes in the dark at either −80°, −20°, 4°, or 20°C. Visual stability was evaluated by taking pictures with a white light screen as background. Activity tests were performed via manual EDS. Ink degradation was evaluated via ion exchange HPLC: Agilent 1260 Infinity II [Thermo Fisher Scientific DNAPac PA200 column (4·250 mm); solvent A, 25 mM tris (pH 8.0); solvent B, 1 M LiCl in Milli-Q; flow rate: 1 ml/min, column temperature: 25°C] equipped with an ultraviolet (UV)–visible detector. Before injection, inks were passed through an Amicon membrane filter (3 kDa, 14,000 rcf, 20 min). Nucleotide quantification was carried out using the automated peak integration of the software. The percentual decrease is based on absolute peak integration (mAU·s), setting the peak area of the nucleotide measured at 0 hours as 100%. The mass of the degradation products was confirmed via HPLC-MS (column HSST2 2.1 mm by 150 mm by 1.8 μm) (Waters, MA, USA). Elution was performed with the following: solvent A, water solution containing 120 mM hexafluoroisopropanol (HFIP) and 5 mM triethylamine (pH 7; adjusted with formic acid), and solvent B, methanol solution containing 120 mM HFIP and 5 mM triethylamine at 40°C. Optical detection was carried out with a photodiode array; mass detection was performed via electrospray ionization (capillary voltage of 2.2 kV, cone voltage of 45 V, desolvation temperature of 400°C, desolvation flow of 500 liters/hour, and cone flow 30 liters/hour).

### Enzymatic DNA synthesis

#### Reagents

Elongation steps in the synthesis cycle used ink Ei-2 (see the “Ink studies” section). Deprotection steps used the buffer (DB) of Hutter *et al.* (*19*), i.e., 0.7 M NaOAc and 1.0 M NaNO_2_ (pH 5.2). The proteinase K wash buffer was proteinase K (0.05 mg ml^−1^) in a 10 mM tris (pH 8.0) solution containing 0.25 mM EDTA and 100 mM NaCl. End-labeling of DNA and capping of primer was performed with a pH 6.6 solution containing 0.5 M cacodylic acid, 1 mM CoCl_2_, 20 μM TdT, 500 μM ddATP (for capping), or 50 μM ddATP-FAM or ddATP-Cy5 (for end-labeling). All solutions were prepared with Milli-Q water. If not otherwise specified, then reagents were purchased from Sigma-Aldrich, Jena Bioscience, AK Scientific, Fisher Chemicals, and New England Biolabs and used without further purification. TdT and dNTP-3′-ONH_2_ were produced internally by DNA Script.

#### Protocols

Inkjet EDS was performed without any optimization of cycle times or processes. The cycle consisted of move-stop printing (see above), incubation (elongation) at 20°C for 10 min, and then slide washing (1 min with proteinase K, 1 min with water, 3 min with DB, and 1 min with water).

End-labeling (10 min at 20°C) was performed after synthesis with 1500 μl of end-labeling solution while incubating the slide at 70% RH. Slides were washed with 1500 μl of DB (2 × 2 min), 1500 μl of Milli-Q water (3 × 1 min), followed by drying with compressed air.

Photocleaving was done by applying an eight-well gasket and adding 150 μl of 0.1× PBS to each well. The substrate was then subjected to UV illumination at λ365 nm (Analytik Jena; 8 W) for 15 min at 20°C. The cleaved oligonucleotides were recovered and then vacuum concentrated (SpeedVac, Eppendorf, Hamburg, Germany) for 45 min at 45°C.

Competitive hybridization used three labeled targets (t-e13-488, t-q4-555, and t-41-647) purchased from Eurogentec (Seraing, Belgium) and diluted to a final concentration of 1 μM each in 5× saline sodium citrate buffer (SSC) containing 0.1% Tween 20. A one-well gasket was applied to the slide and the surface incubated with 1300 μl of hybridization solution for 15 min at 60°C followed by 2 hours at 20°C (300 rpm) in a humid chamber at 70% RH. This was followed by slide washing at 20°C with 1300 μl of 5× SSC, 1300 μl of 4× SSC, 1300 μl of 2.5× SSC, 1300 μl of 1× SSC, and 5 × 1300 μl in Milli-Q water and then immediate drying under compressed air. Synthesized 50-mer *XYZ* probes were hybridized in a stepwise manner because of the occurrence of t-e13-488 and t-q4-555 quenching (through fluorescence resonance energy transfer) by t-q41-647 (see fig. S16).

Reverse transcription was performed by adapting the protocol from New England Biolabs. Hybridization of mRNA onto synthesized poly(T) oligomers (see fig. S2) in ribonuclease-free water ([mRNA] = 0.6 μM, dNTP = 1 mM, 65°C for 5 min followed by rapid cooling for 3 min at 0°C) was followed by reverse transcription (1 hour, 42°C) in 1× ProtoScript II buffer with ProtoScript II reverse transcription (10 U/μl) and 10 mM dithiothreitol. Deactivation of the enzyme was carried out following the proteinase K treatment described above. Dehybridization of mRNA was done by consecutive washes at 60°C with 3 M NaCl for 30 min, 5× SSC for 5 min, 4× SSC for 1 min, 2.5× SSC for 1 min, 1× SSC for 1 min, and Milli-Q for 5 min. cDNA products were end-labeled and cleaved as previously described.

#### Analysis

The arrays were imaged with a Zeiss Axiolmager Z2 epifluorescence microscope (×5 magnification, channels, GFP λex 470, DsRed λex 555, and Cy5 λex 625) equipped with an automatic stage. Light-emitting diode intensities and exposure times were adjusted to the fluorophore used and the nature of the experiment.

Image analysis was performed with ImageJ (bio-format plugin) after defining boundary conditions. Diameters in Fig. 2 (C and F) were calculated by including all spots of the section (i.e., ≈1200). The values are the mean diameters from analyzing two slides, i.e., ≈2400 spots. The error bars are ±SD. The diameters reported in Fig. 5 (C and D) are the mean values ± SD from the measurement of 1200 spots in each section. Measurement was performed on a single slide.

Cleaved oligos were mixed (1:1) with Brilliant Blue and gel loading buffer and loaded onto a polyacrylamide gel [30 ml of polyacrylamide, 80 μl of ammonium persulfate, and 28 μl of TEMED (N,N,N′,N′-tetramethylethylenediamine)] and electrophoresed at 1500 V (EV3330 Consort). The gel was then imaged with the Amersham Typhoon Biomolecular Imager, and the images were analyzed with Bio-Rad Image Lab software with gamma, high, and low parameters set to 1.8, 800, and 150, respectively. The same Typhoon instrument was used to image the whole slide in fig. S3.

## Notes

View/request a protocol for this paper from *Bio-protocol*.
