# Supplementary material for: Multiplex enzymatic synthesis of DNA with single-base resolution
Source: Sci Adv. 2023 Jul 7;9(27):eadi0263. doi: 10.1126/sciadv.adi0263 (PMC10328407; doi:10.1126/sciadv.adi0263)
Supplement: Supplementary file 1 — Figs. S1 to S20 [file sciadv.adi0263_sm.pdf]

Science Advances
AAAS

Supplementary Materials for

Multiplex enzymatic synthesis of DNA with single-base resolution

Damiano Verardo *et al.*

Corresponding author: Adrian Horgan, adrian.horgan@dnascript.com

Sci. Adv. **9**, eadi0263 (2023)
DOI: 10.1126/sciadv.adi0263

This PDF file includes:

Figs. S1 to S20

**Citation**	**Enzyme**	**Template -free**	**Reversible terminator**	**Deblock method**	**Spatial control**	**Sites of synthesis**	**Single base extension**	**Cycle efficiency**	**Length**	**Comments**
Mathews et al., (*15*)	Unmodified TdT	Yes	3-O-nitrobenzyl	Light (365 nm)	**No**	**-**	**Yes**	**Not reported**	**4 bases**	TdT was not adapted to the steric bulk of the 3’-nitrobenzyl group
Palluk et al., (*13*)	TdT-dNTP conjugate	Yes	-	Light (365 nm)	**No**	**-**	**Yes**	**<98%**	**10 bases**	Scar left on the nucleobase
Hoff et al., (*2*)	Modified 9°N enzyme	No	3’-O-azidomethyl	TCEP (aq.)	**No**	**-**	**Yes**	**98%**	**20 bases**	Not a template-free (e.g., TdT) method
Lee et al., (*25*)	TdT and apyrase	Yes	-	-	**No**	**-**	**No**	**-**		Competition used to prevent homopolymer formation
Lee at al., (*26*)	Unmodified TdT	Yes	-	-	**Yes**	**12**	**No**	**-**	**8 cycles**	First demonstration of EDS multiplexing (no single base control)
Lu et al., (*22*)	Modified TdT	Yes	3’-O-aminoxy	Sodium nitrite (aq.)	**No**	**-**	**Yes**	**98.7%**	**10 bases**	
**This work**	Modified TdT	Yes	3’-O-aminoxy	Sodium nitrite (aq.)	**Yes**	**>20,000**	**Yes**	**>99%**	**50 bases**	First demonstration of EDS multiplexing and single base control

Fig. S1. Enzymatic DNA synthesis strategies

The table above summarizes EDS progress reported in the literature and the approaches taken.

Name	Sequence (5’-3’)	Moiety	Role	Length *	%GC **	Tm / °C **	Fig.
unlabP'	*TTTTTTTTTTPCTTTTTTTTTTTTTTTTTTT TTT*	5’DBCO-TEG	Primer	33	-	48.5	
labP'	*TTTTTTTTTTPCTTTTPCTTTTTTTTTTTTTT TTT*	5’DBCO-TEG, 3’FAMdT	Primer	31	-	47.3	2B/C/D
labP’’	*TTTTTTTTTTFAMTTTTTTTTTTPCGCTGT TTCGCGTGACATTCTAAATACGGATGTG GC*	5’DBCO-TEG, FAMdT	Primer	56	30.4	65.1	5F
unlabP’’	TTTTTTTT	-	Probe	21	-	0.0	3C/D
poly(T)$_1$	T	-	Probe	1	-	0.0	4C
poly(T)$_2$	TT	-	Probe	2	-	0.0	4C
poly(T)$_3$	TTT	-	Probe	3	-	0.0	4C
poly(T)$_4$	TTTT	-	Probe	4	-	0.0	4C
poly(T)$_5$	TTTTT	-	Probe	5	-	0.0	4C
poly(T)$_6$	TTTTTT	-	Probe	6	-	0.0	4C
poly(T)$_7$	TTTTTTT	-	Probe	7	-	0.0	4C
poly(T)$_8$	TTTTTTTT	-	Probe	8	-	0.0	4C
e13	GAACTTCAACTTCAACGGCCT	-	Probe	21	47.6	55.7	4D, 5A
q4	GTCTCTGCGGAGGAAGACACT	-	Probe	21	57.1	58.6	4D, 5A
q41	GCTGTTTCGCGTGACATTCTA	-	Probe	21	47.6	55.6	4D, 5A
t-e13	*TAGGCCGTTGAAGTTGAAGTTC*	5’Alexa488	Target	22	45.5	55.2	4D, 5A/B
t-q4	*TAGTGTCTTCCTCCGCAGAGAC*	5’Alexa555	Target	22	55.4	55.8	4D, 5A/B
t-q41	*TTAGAATGTCACGCGAAACAGC*	5’Atto647N	Target	22	45.5	55.9	4D, 5A/B
e13-poly(T)n+8-q4	GAACTTCAACTTCAACGGCCTTTTTTTT TTGTCTCTGCGGAGGAAGACACT	-	Probe	50	44.0	67.7	5B
q4-poly(T)n+8-q41	GTCTCTGCGGAGGAAGACACTTTTTTT TTGCTGTTTCGCGTGACATTCTA	-	Probe	50	44.0	67.6	5B
q41-poly(T)n+8-e13	GCTGTTTCGCGTGACATTCTATTTTTTTT TGAACTTCAACTTCAACGGCCT	-	Probe	50	40.0	66.7	5B
e13$_{D20,21}$	GAACTTCAACTTCAACGGC~~CT~~	-	Probe	19	47.4	51.9	5C
e13$_{D18,19}$	GAACTTCAACTTCAACG~~G-C~~CT	-	Probe	19	42.1	50.7	5C
e13$_{D16,17}$	GAACTTCAACTTCAA~~CG~~GCCT	-	Probe	19	42.1	50.2	5C
e13$_{D14,15}$	GAACTTCAACTTC~~AA~~-CGGCCT	-	Probe	19	52.6	55.2	5C
e13$_{D12,13}$	GAACTTCAACT~~TC~~AACGGCCT	-	Probe	19	47.4	52.4	5C
e13$_{S19-21}$	GAA CTT CAA CTT CAA CGG **GAC**	-	Probe	21	47.6	54	5D
e13$_{S13-15,19-21}$	GAA CTT CAA CTT **ATC** CGG **GAC**	-	Probe	21	47.6	53.1	5D
e13$_{S16-21}$	GAA CTT CAA CTT CAA **GCT GAC**	-	Probe	21	42.9	51.8	5D
e13$_{S13-21}$	GAA CTT CAA CTT **ATC GCT GAC**	-	Probe	21	42.9	51.4	5D
e13$_{S16-21}$	GAA CTT CAA CTT CAA **GAC ACT**	-	Probe	21	38.1	50.5	5D

poly(T)$_5$	TTTTTTTTTT	-	Probe	10	-	11.3	5F
poly(T)$_{10}$	TTTTTTTTTT	-	Probe	10	-	11.3	5F
poly(T)$_{20}$	TTTTTTTTTTTTTTTTTTTT	-	Probe	20	-	37.3	5F
poly(T)$_{30}$	TTTTTTTTTTTTTTTTTTTTTTTTTTTTTT	-	Probe	30	-	46.7	5F
mRNA	*UACACGUUGUCUAUCGCCUUAAAAAAA AAAAAAAAAAAAAAAAAAAAAAA*	-	Template	50	18.0	60.1	5F
cDNA(T)$_{10\text{-}30}$	*TTTTTTTTTTTTTTTTTTTTTTTTTTTTTTAA GGCGATAGACAACGTGTAT*	3'FAMdT	cDNA	51	17.6	60.2	5F

Fig. S2. Sequences and properties of DNA primers, synthesized probes, complementary targets and mRNA

Italics mean not synthesized. PC = photocleavable group. * Length does not include primer length. When photocleaved the product will be 23 bases longer. Underlined means can be removed from the product by cleaving enzymatically (*58*). ** Not including the primer. Strikethrough bases are deletion from e13 sequence. Bold font bases are substitutions to e13 sequence. Calculated by IDT's Oligoanalyzer™.

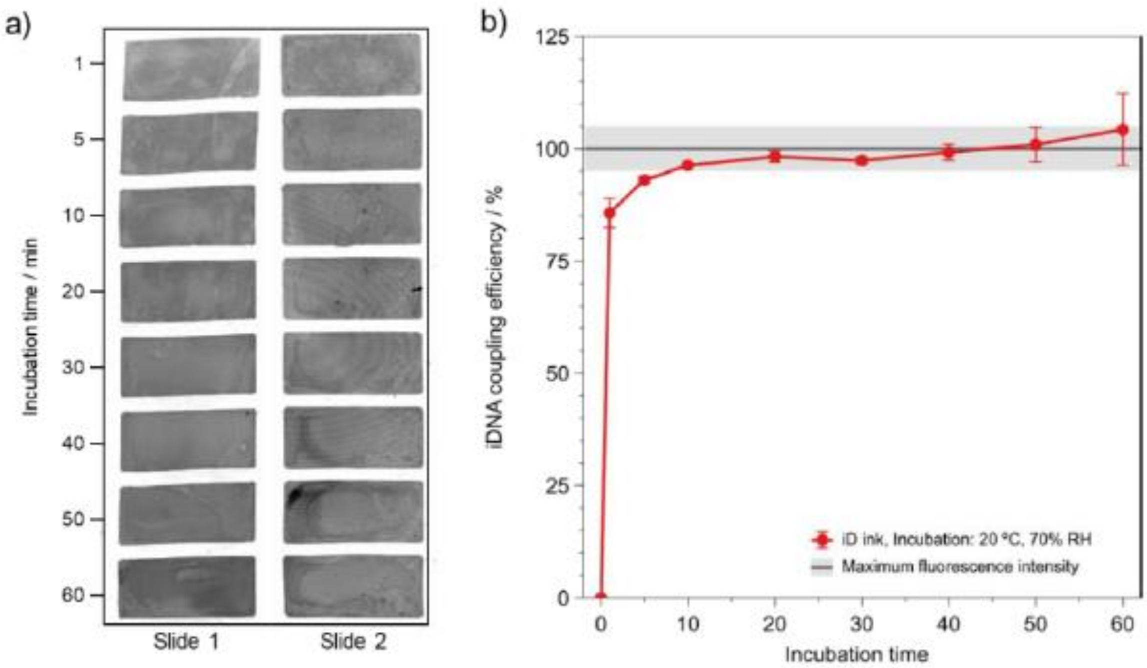

Fig. S3. DNA immobilization using Click chemistry

The graph above shows the coupling of a FAM-labelled primer (see ‘labP’ in **S2**) to a 3’azide microscope slide. Coupling is ~85% complete in 1 min. Across an entire slide the signal variation was <4%. For the composition of the coupling buffer see Materials and Methods.

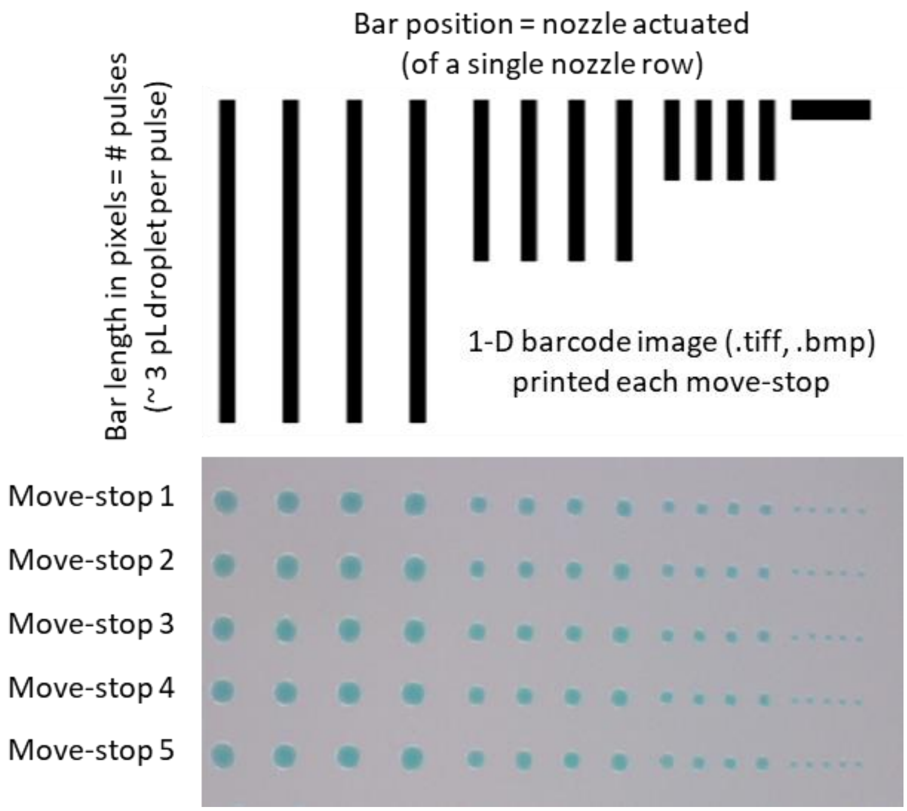

Fig. S4. 1-D barcode for move-stop-print method

The figure above shows a schematic of a 1-D barcode. When printed the barcode image defines which nozzle(s) fire and how many times the nozzles fire after each move-stop. Only nozzles in 1 of the 2 nozzle rows are used (see **S6**). The accompanying image was captured automatically by camera under IPA control and shows the different droplet (wet) sizes generated using the barcode. The barcode was printed 5 times and the ink contained an enzyme-compatible dye (Fast Green) to improve contrast.

Resolution (dpi)	Spot-spot distance (mm)	Max spots/slide Square array	Max spots/slide Hexagonal array
50	0.5075	7.2k	8.4k
60	0.4229	10.5k	12.1k
75	0.3383	16.4k	18.9k
100	0.2537	29.1k	33.6k
150	0.1692	65.5k	75.6k
300	0.0846	262k	303k
600	0.0423	1.05M	1.21M

Fig. S5. Spot density

The table above gives the maximum number of synthesis sites for a standard 75 x 25 mm microscope slide based on the specification of the printhead. The printhead gives a print swathe 33.8 mm wide and has 800 nozzles in two interleaved rows to afford 600 dpi native resolution. Only nozzles from the central 70% are used for printing onto the glass microscope slide. Within a row the nozzles are 1/300 th inch apart. The move-stop-print method uses only the nozzles of a single row i.e., gives 300 dpi. In this work, a print resolution of 75 dpi was chosen for synthesis. With a square lattice 75 dpi gives 16,400 sites per slide. Not all of the slide was printed on to allow the application of gaskets to hold liquids necessary for DNA hybridization and DNA cleaving. Considering the gasket, the number of synthesis sites is closer to 10,560 spots.

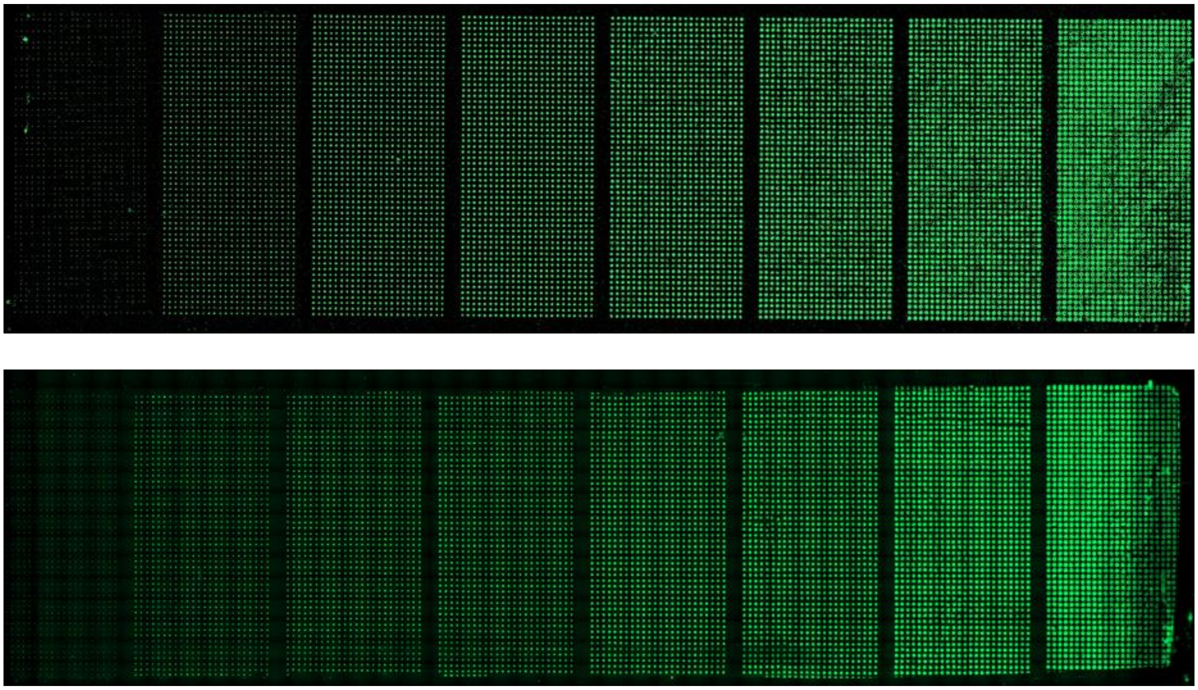

Fig. S6. Spot (dry) size (see Fig. 2B)

The figure above shows fluorescence microscope images for: (top to bottom) DNA ink on azide slide after coupling and automated washing and drying; Elongation ink (Ei-2) after incubation, manual capping (see Materials and Methods), automated washing and drying, manual end-labelling (see Materials and Methods).

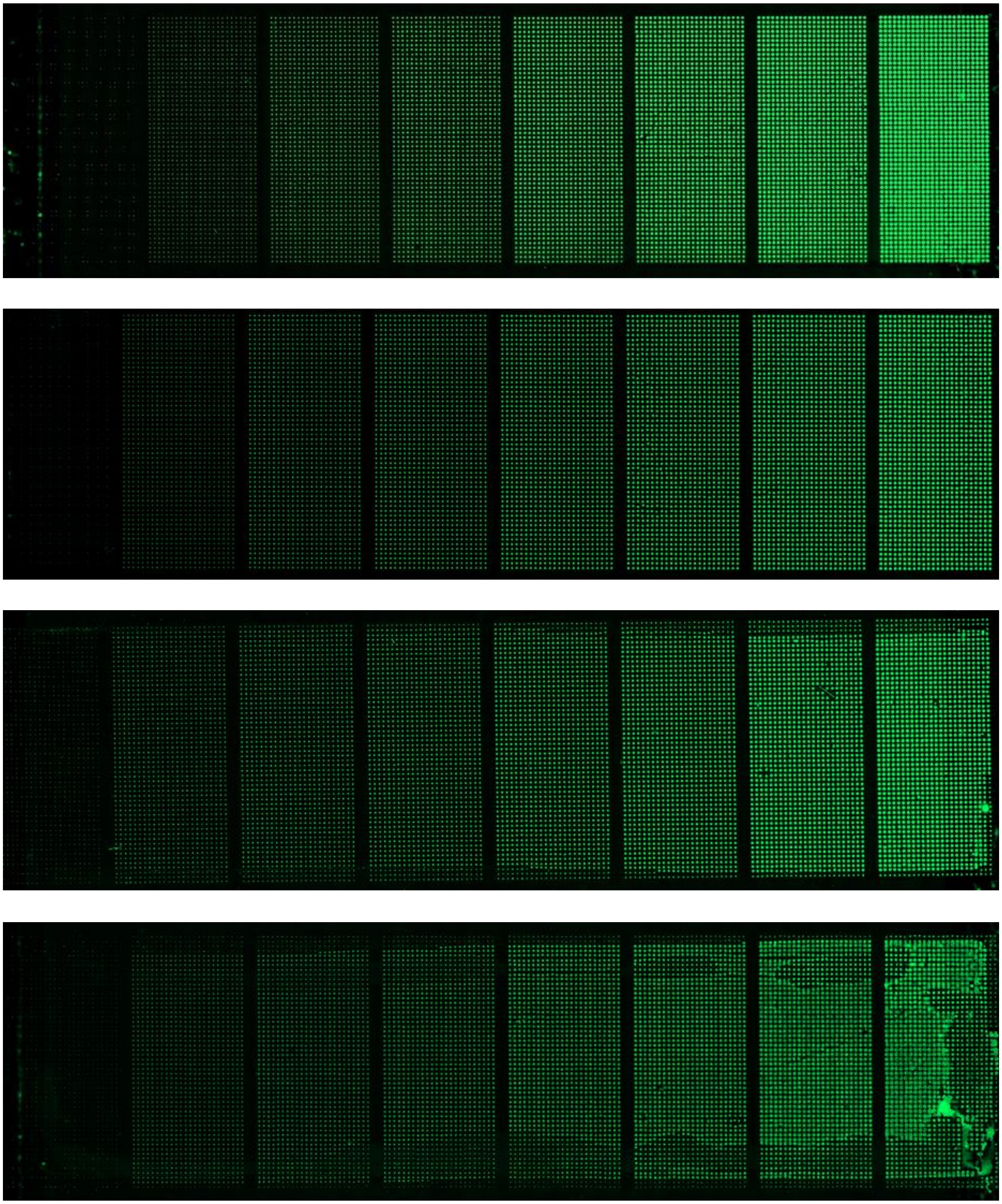

Fig. S7. Droplet (wet) size (see Fig. 2C)

The figure above shows fluorescence microscope images for: (top to bottom) DNA ink on azide slide; elongation ink (Ei-2) on azide slide; elongation ink (Ei-2) on DNA slide; DNA ink on DNA slide. 2 slides were printed for each case. Only 1 of each case is shown for example purposes.

Elongation ink contained 50 µM fluorescein. All slides were imaged just after printing (wet) without further treatment.

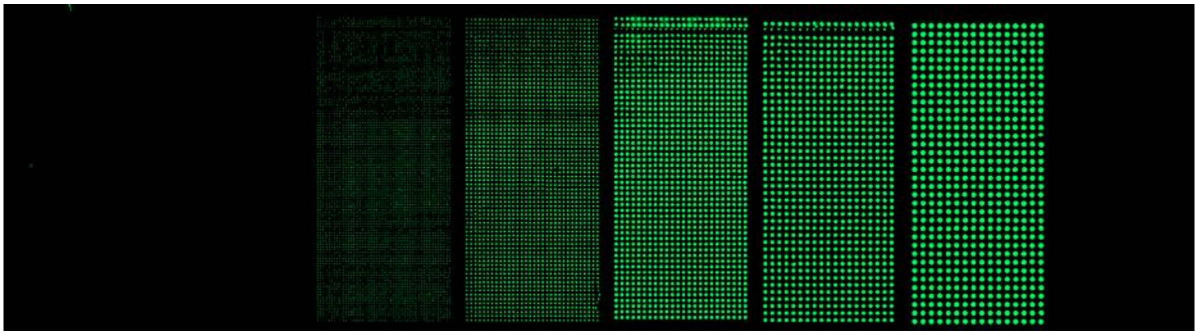

Fig. S8. Pitch (see Fig. 2D)

The whole slide epifluorescence microscope image is shown above for the test done to see if droplet (wet) size can be varied at the same time as pitch by printing barcode images with different bar spacing and bar lengths (see S4).

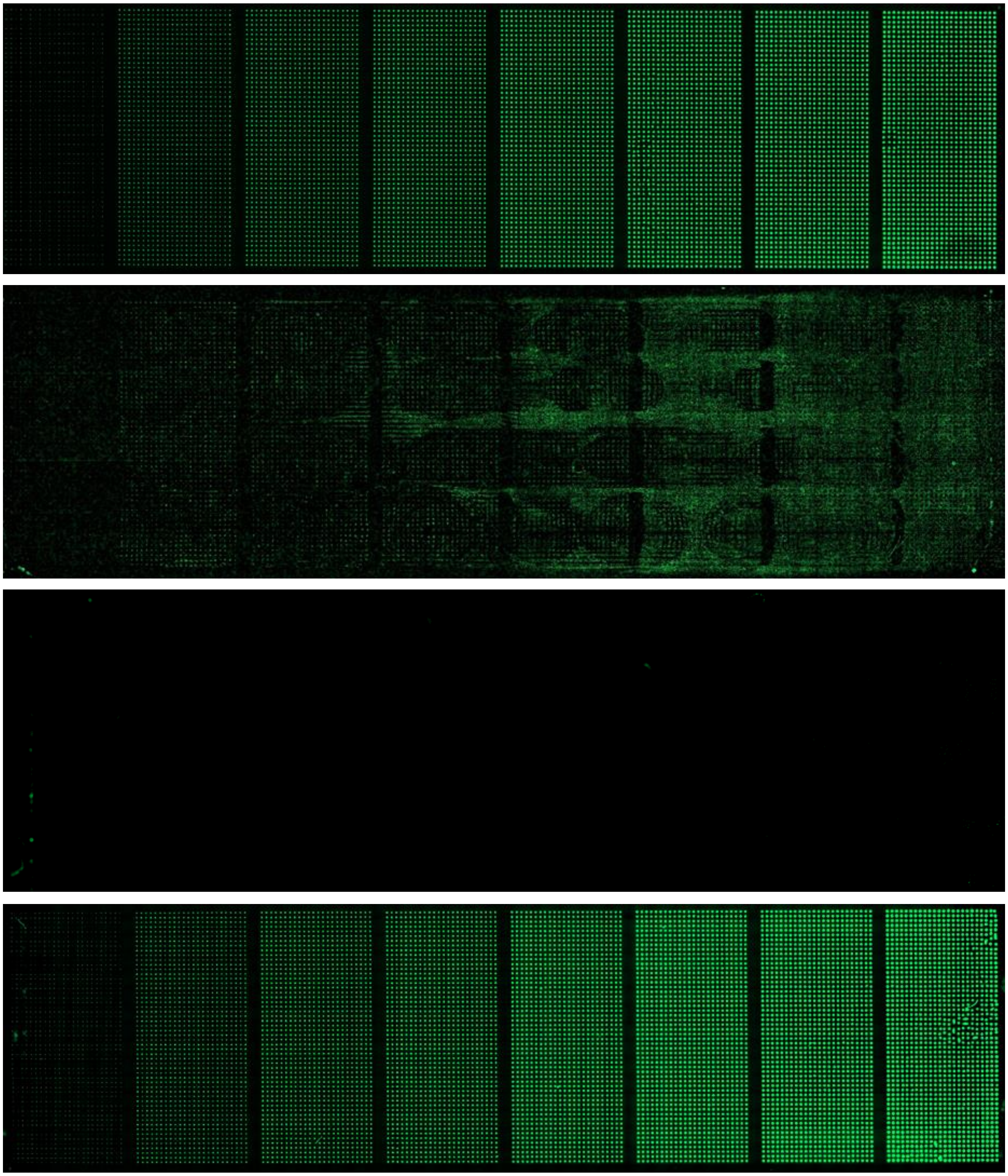

AZIDE

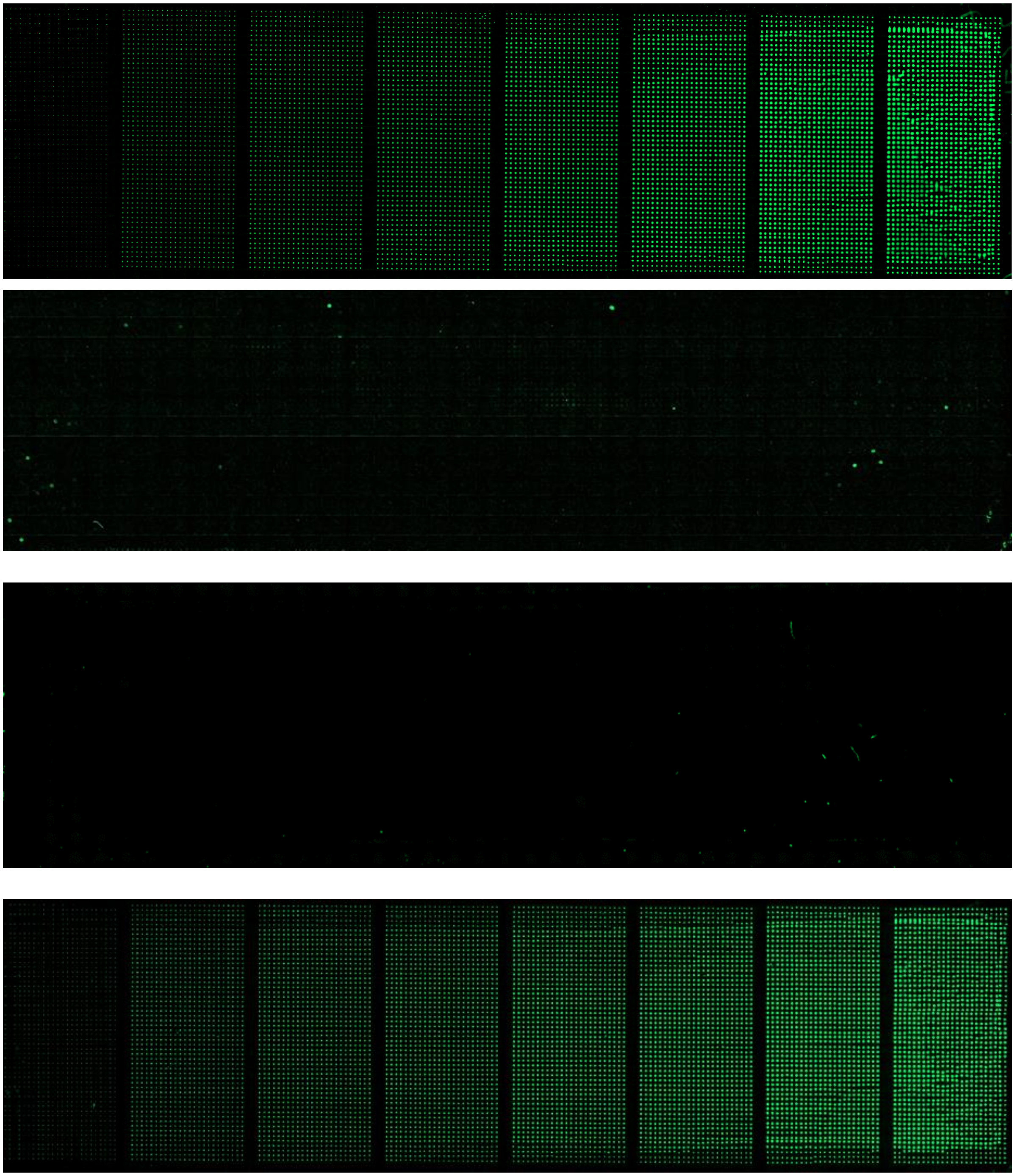

DNA

Fig. S9. Protein adsorption (see Fig. 2E/2F)

The above images are the whole slide images for the GFP-TdT adsorption test with the azide and DNA slides. The order is i) after printing, ii) after automated deprotection, water washing and drying, iii) after automated proteinase K treatment, deprotection, water washing and drying, iv) after printing elongation ink.

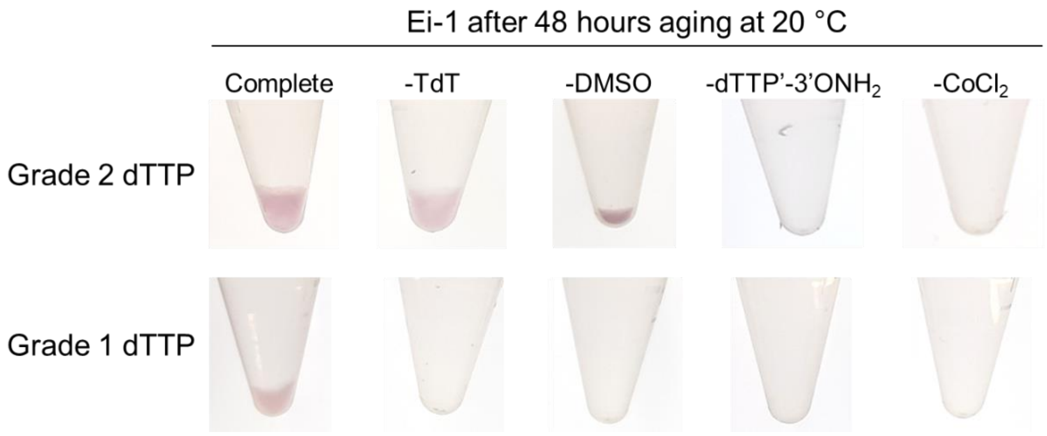

Fig. S10. Precipitation level versus purity of nucleotide

The image above shows how precipitation of Ei-1 ink is different for two grades of dTTP-3'ONH$_2$. Photos were taken of samples after 48 hours aging at 20 °C. Grade 1 dTTP-3'ONH$_2$ is purer (> 95% purity) and does not contain pyrophosphates and polyphosphates. Ink formulations containing grade 1 dTTP-3'ONH$_2$ exhibit less precipitate and no precipitate upon removing single components.

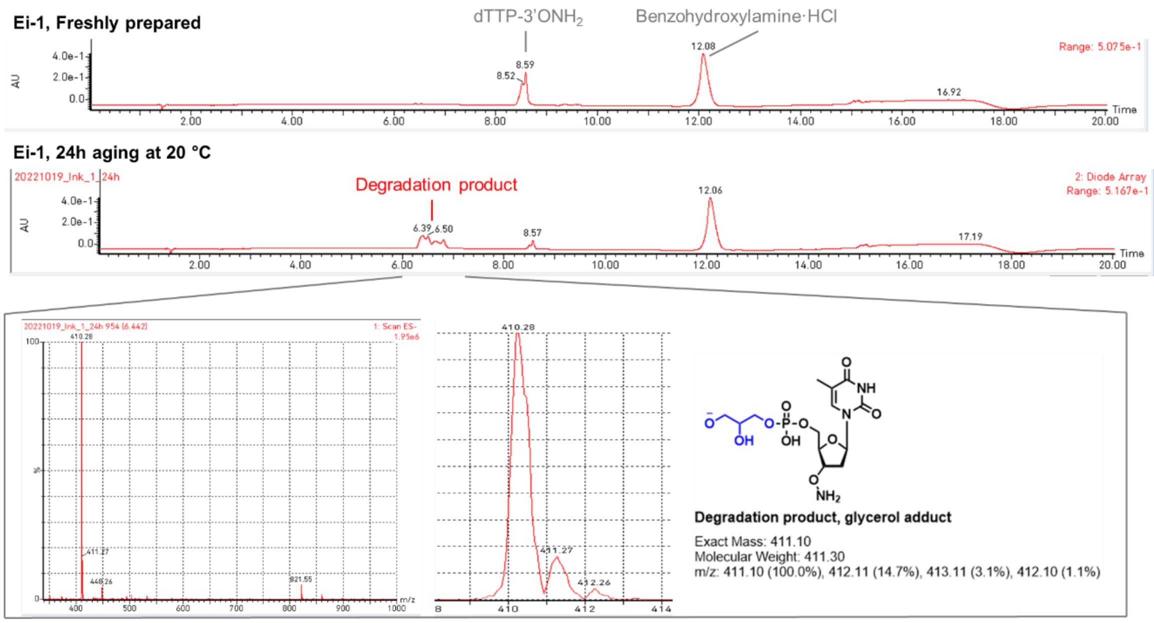

Fig. S11. Glycerol adduct (see 'Ink Formulation')

In the image above are shown the HPLC-UV chromatograms of fresh Ei-1 ink (top) and aged (24 hours at 20 °C) Ei-1 ink (middle). Also shown is the LCMS spectrum (bottom, left) of the product resulting from degradation of the 3'blocked nucleotide. It is posited to be a glycerol adduct (bottom, right). LCMS of ink Ei-2 yielded an analogous result (data not reported).

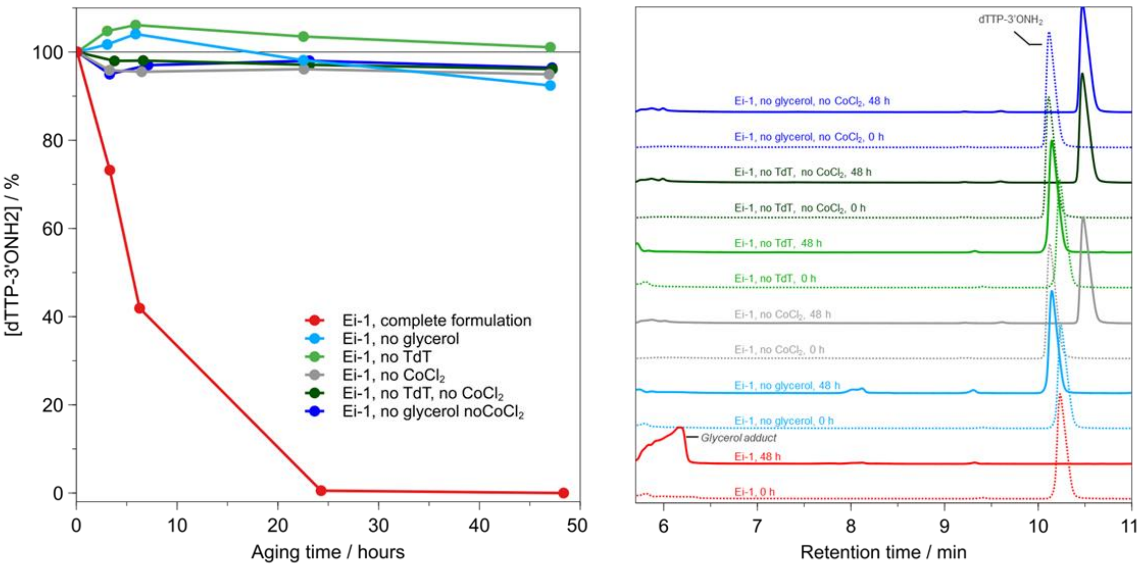

Fig. S12. Non-hydrolysis pathway (see 'Ink Formulation')

Further support for the non-hydrolysis pathway comes from HPLC-UV data for Ei-1 ink with different components removed. The left image above shows the percentage decrease in [dTTP-3'ONH_2]. The right image above shows the corresponding chromatograms for fresh and aged (48 hours at 20 °C) inks. Ei-1 ink: with all components (red line); in absence of TdT (green line); in the absence of $CoCl_2$ (grey line); in absence of glycerol (blue line).

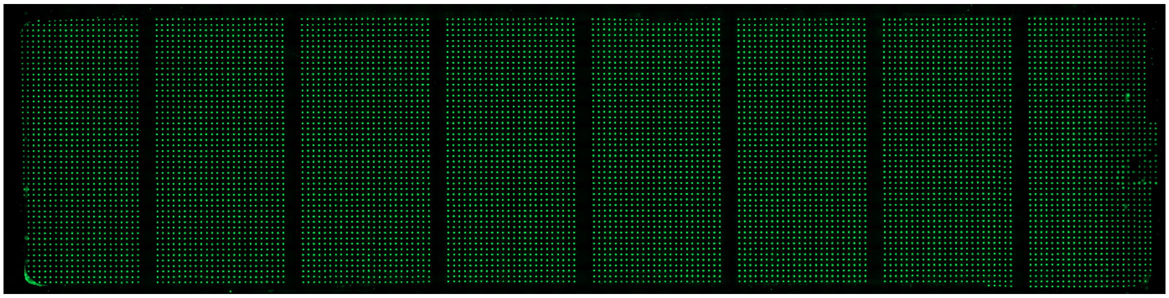

Fig. S13. Single base extension of primer (see Fig. 4C)

The figure above is the fluorescence microscope image of the whole slide taken after automated 8-cycle inkjet poly(T) synthesis and manual end-labelling with ddATP-FAM (see Materials and Methods). The number of cycles was reduced from the farthest left section (8 cycles) to the farthest right section (1 cycle). Approximately 1,000 oligo spots were cleaved per section and analyzed by gel electrophoresis (see Materials and Methods).

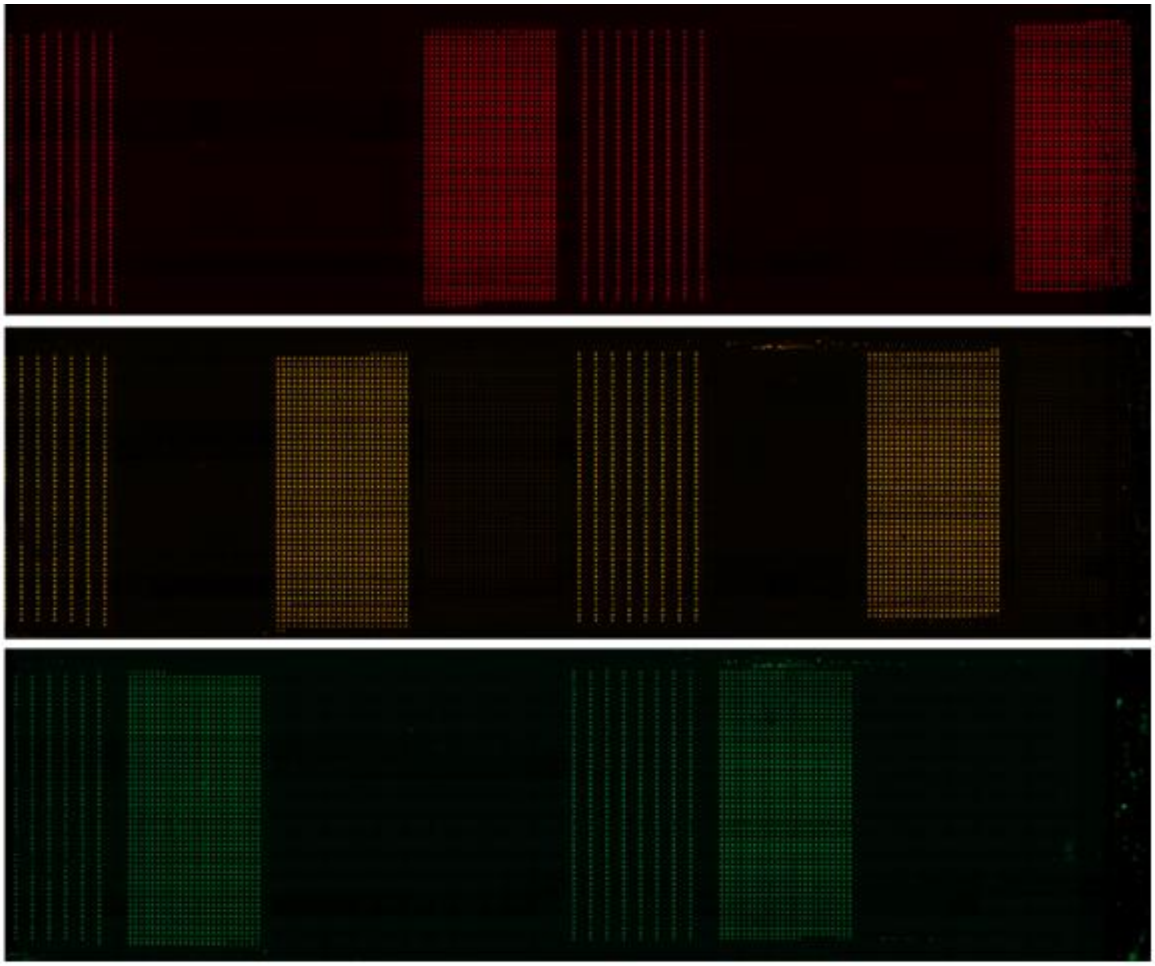

Fig. S14. Spatial EDS using inkjet dispensing (see Fig. 4D)

The fluorescence microscope images above show the signal from the 3 channels (top to bottom: Channel 1 = Cy5; Channel 2 = DsRed; Channel 3 = EGFP). No cross-hybridization is apparent. For example, consider the hybridization of t-q41 (see **S2**) to the inkjet synthesized q4 probe. No red signal corresponding to t-q41 is seen in the sections corresponding to q4 (middle image, sections 3 & 7 from left to right).

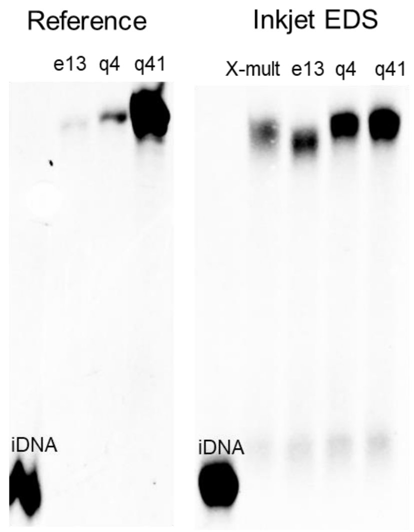

Sequence	e13	q4	q41
Purity %	77	80	80
Cycle efficiency %	98.8	98.9	98.9

Fig. S15. Spatial EDS (see Fig. 4D)

Electrophoresis gel of 3’ddATP-FAM labeled e13, q4, and q41 probes (see S2) synthesized in S14. Purity is calculated from the lane profile and integration of the bands. Cycle efficiency is calculated as $\sqrt[1/21]{Purity}$.

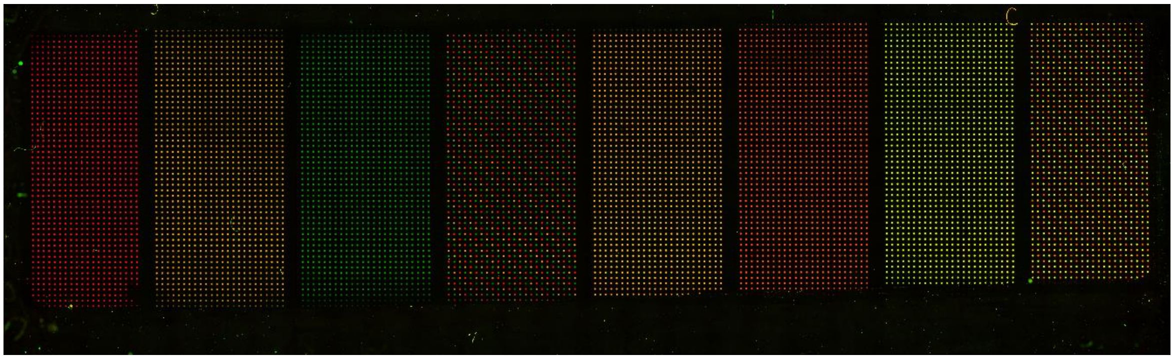

COMBINED

Hybridization with t-q4-555; single channel image (Cy3)

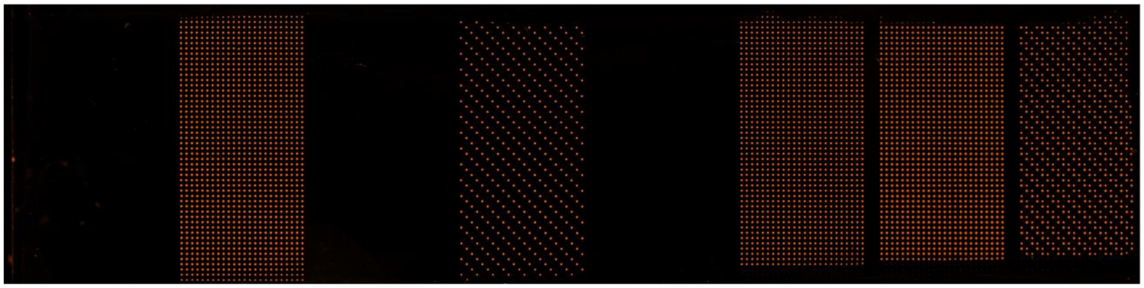

Hybridization with t-e13-488 and t-q4-555; single channel image (EGFP)

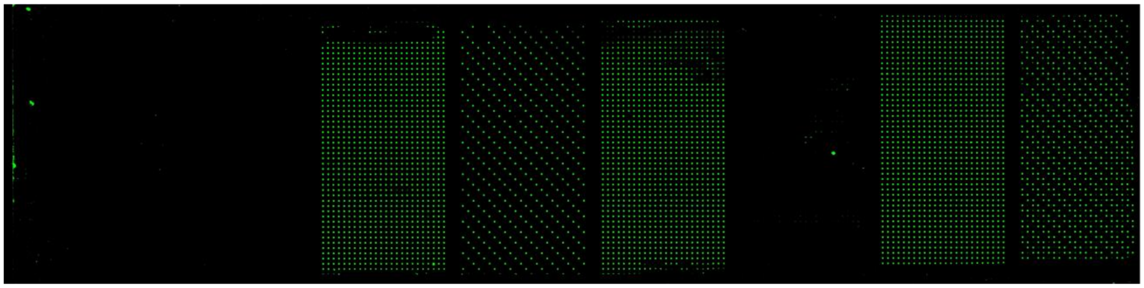

Hybridization with t-q41-647, t-e13-488 and t-q4-555; single channel image (Cy5)

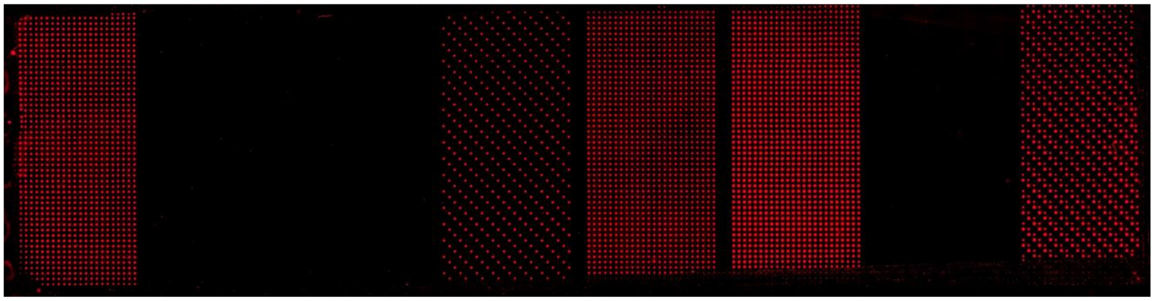

Hybridization with t-q41-647, t-e13-488 and t-q4-555; single channel image (Cy3)

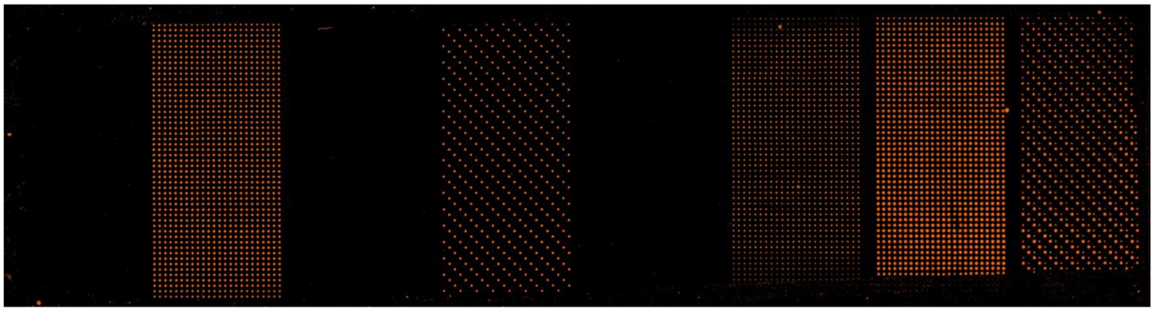

Hybridization with t-q41-647, t-e13-488 and t-q4-555; single channel image (EGFP)

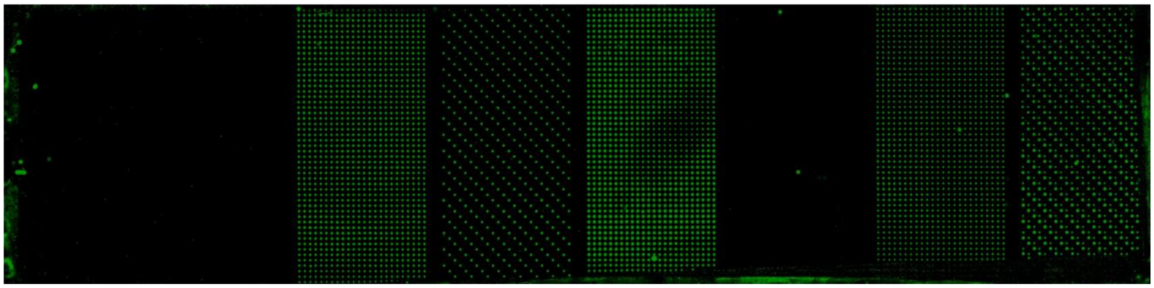

SINGLE CHANNEL

Fig. S16. XYZ multiplexing (see Fig. 5B)

The combined 3 channel fluorescence microscope image above (top) shows the whole slide after stepwise hybridization first with t-q4-555 (Cy3 channel), then with t-e13-488 (EGFP channel) and t-q4-555 (Cy3 channel), and then with t-e13-488 (EGFP channel) and t-q4-555 (Cy3 channel) and t-q41-647 (Cy5 channel) (from left to right: q41, q4, e13, q41/q4/e13 (XY pattern), q41-p(T)n+8-e13, q4-p(T)n+8-q41, e13-p(T)n+8-q4, q41-p(T)n+8-e13/q4-p(T)n+8-q41/e13-p(T)n+8-q4 (XYZ patten). See **S2** for probe sequences.

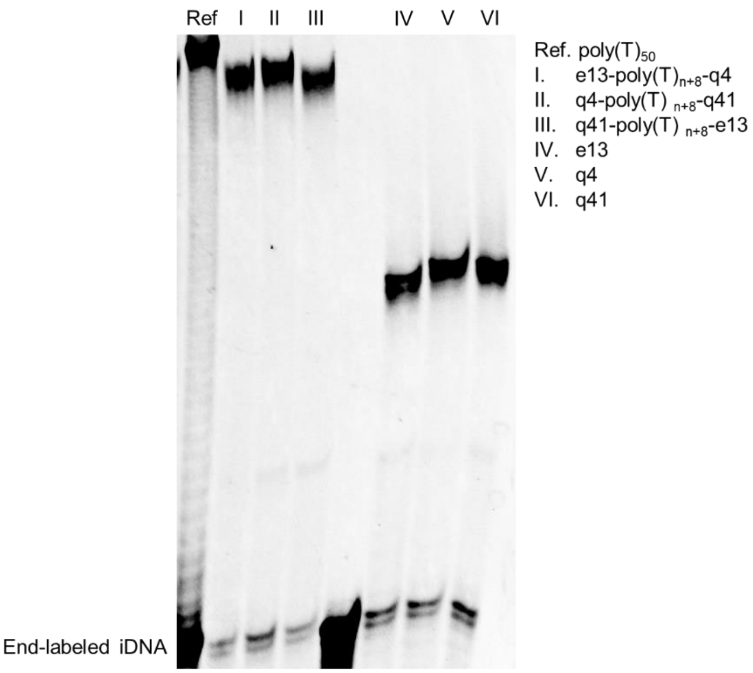

Fig. S17. XYZ multiplexing (see Fig. 5B)

Electrophoresis gel of 3'ddATP-FAM labeled 50mers and 21mers (see **S2**) synthesized via inkjet in S16.

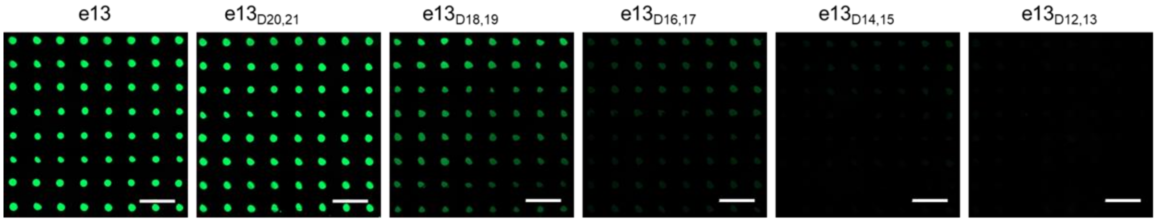

Fig. S18. Double deletions (see Fig. 5C)

The decrease in fluorescence intensity observed as double deletions are progressively moved from the 3'terminus towards the middle of the e13 probe (see **S2**). Cropped images after hybridization with t-e13-488. Brightness and contrast values are the same for the 6 images. Scale bar = 500 µm.

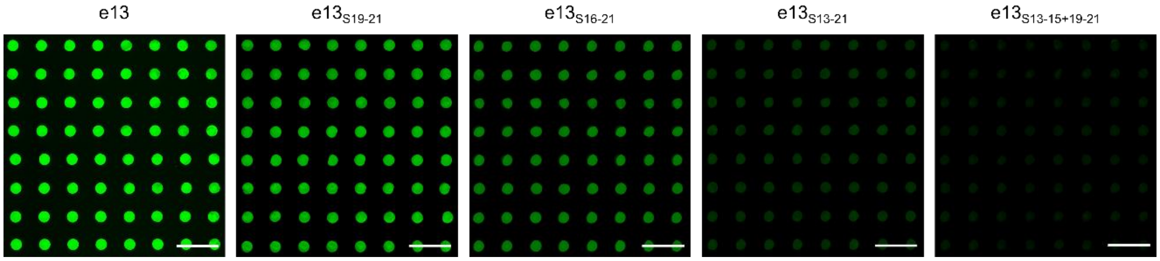

Fig. S19. Substitutions (see Fig. 5D)

The decrease in fluorescence intensity observed as progressively more mutations are incorporated in the e13 probe sequence (see **S2**). Cropped images after hybridization with t-e13-488. Brightness and contrast values are the same for the 6 images. Scale bar = 500 µm.

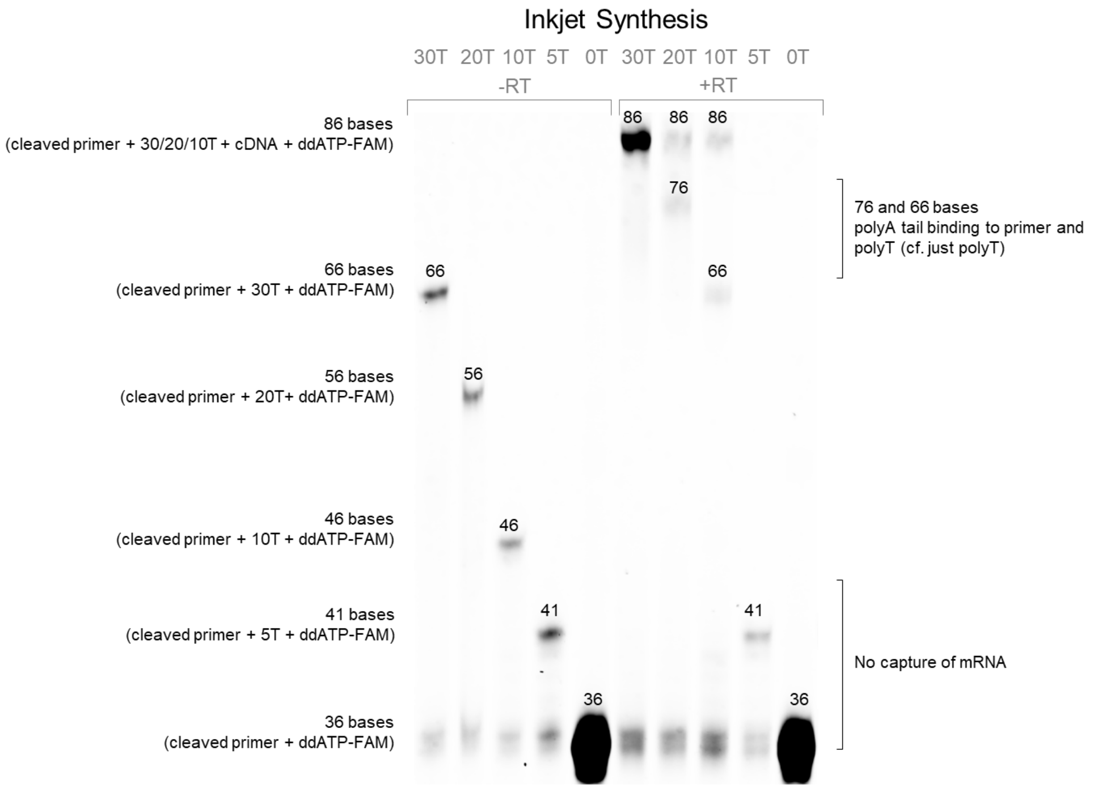

Fig. S20. mRNA capture and reverse transcription (see Fig. 5F)

The presence of a single, dark DNA band with length 86 bases in the gel above shows that capture of mRNA (see **S2**) is more efficient when the polyT tail has 30 bases. With less than 30 bases capture also involves partial overlap of the mRNA's polyA$_{(30)}$ tail with the DNA primer and results in two bands. However, the RNA information is retained. Below 10 bases the mRNA capture is negligible.
